# Supplementary material for: Plasmodium vivax chloroquine resistance links to pvcrt transcription in a genetic cross
Source: Nat Commun. 2019 Sep 20;10:4300. doi: 10.1038/s41467-019-12256-9 (PMC6754410; doi:10.1038/s41467-019-12256-9)
Supplement: Supplementary file 1 — Supplementary Information [file 41467_2019_12256_MOESM1_ESM.pdf]

Supplementary Information

Plasmodium vivax chloroquine resistance links to *pvcrt* transcription in a genetic cross

Sá *et al*.

Table of Contents

Supplementary Figure 1. Representative *P. vivax* NIH-1993 and AMRU-I responses to CQ treatments in *Aotus* or *Saimiri* monkeys…………………………………………………….**page 4**

Supplementary Figure 2. Chimpanzee *P. vivax* parental lines infection and cross-fertilization in *Anopheles* mosquitoes………………………………………………………………….**page 6**

Supplementary Figure 3. Chart of *Aotus* and *Saimiri* monkey inoculations with NIH-1993 S×R progeny for pooled selection and linkage group analysis…………………………………...**page 8**

Supplementary Figure 4. Recrudescences after CQ treatment of *Aotus* and *Saimiri* monkeys infected with NIH-1993 S×R progeny pools PP1 or PP2……………………………..….**page 10**

Supplementary Figure 5. Map showing chromosomal positions of the 37 microsatellite markers that distinguish parental polymorphisms in the NIH-1993 S×R cross……..………………**page 12**

Supplementary Figure 6. Linkage group selection (LGS) analysis of chromosome 5 by targeted genomic sequencing……………………………………………………………………..**page 13**

Supplementary Figure 7. Aligned sequences from the flanking regions, introns and exons of *pvcrt* in NIH-1993-S, NIH-1993-R and Salvador-I reference parasites………………….…....**page 14**

Supplementary Table 1. *P. vivax* dihydrofolate reductase sequence polymorphisms in the NIH-1993 and Salvador-I lines…………………………………………………………..…**page 18**

Supplementary Table 2. Parasitemia (%) development of NIH-1993 and AMRU-I infections in *Aotus* and *Saimiri* monkeys……………………………………………………………...**page 19**

Supplementary Table 3. Development of *P. vivax* chimpanzee infection after blood-stage parasite inoculation………………………………………………………………………….....…**page 22**

Supplementary Table 4. Genotypes of *P. vivax* lines inoculated into a chimpanzee……...**page 24**

Supplementary Table 5. *P. vivax* infections of *Anopheles* mosquitoes fed on chimpanzee blood……………………………………………………………………….……….**page 25**

Supplementary Table 6. Development of *P. vivax* in chimpanzee after NIH-1993 S×R recombinant sporozoite inoculation……………………………………………….……..**page 26**

Supplementary Table 7. Parasitemia (%) development of NIH-1993 S×R progeny in *Aotus* and *Saimiri* monkeys……………………………………………………………….…...**page 27**

Supplementary Table 8. Chloroquine plasma concentrations in *Aotus* and *Saimiri*….…..**page 32**

Supplementary Table 9. Ratios of parental microsatellite intensities after and before CQ treatment, from CQ-treated monkeys infected with NIH-1993 S×R progeny…………..….**page 34**

Supplementary Table 10. *P. vivax* single nucleotide polymorphisms in a CQ-selected 76 kb region of chromosome 1……………………………………………………………….….**page 36**

Supplementary Table 11. Relative *pvcrt* transcript levels in *Aotus* and *Saimiri* infected with NIH-1993 S×R parasites……………………………………………………………………**page 38**

Supplementary Table 12. Liquid chromatography multiple reaction monitoring (LC-MRM) parameters for the PvCRT selected peptide……………………………………………..**page 40**

Supplementary Table 13. PvCRT protein levels as determined by liquid chromatography multiple reaction monitoring (LC-MRM) analyses……………………………….…..**page 41**

Supplementary Table 14. Estimations of PvCRT concentration in magnetically-purified *P. vivax*-infected red blood cells……………………………………………………………**page 42**

Supplementary Table 15. Repeat sequences in *pvcrt* 5’-UTR and intron 9 that are found in regulatory elements of other species…………………………………………………….**page 43**

Supplementary Table 16. Sequences of oligonucleotide primers used in this study….**page 45**

Supplementary References. ………………………………………………………...**page 48**

Supplementary Figure 1

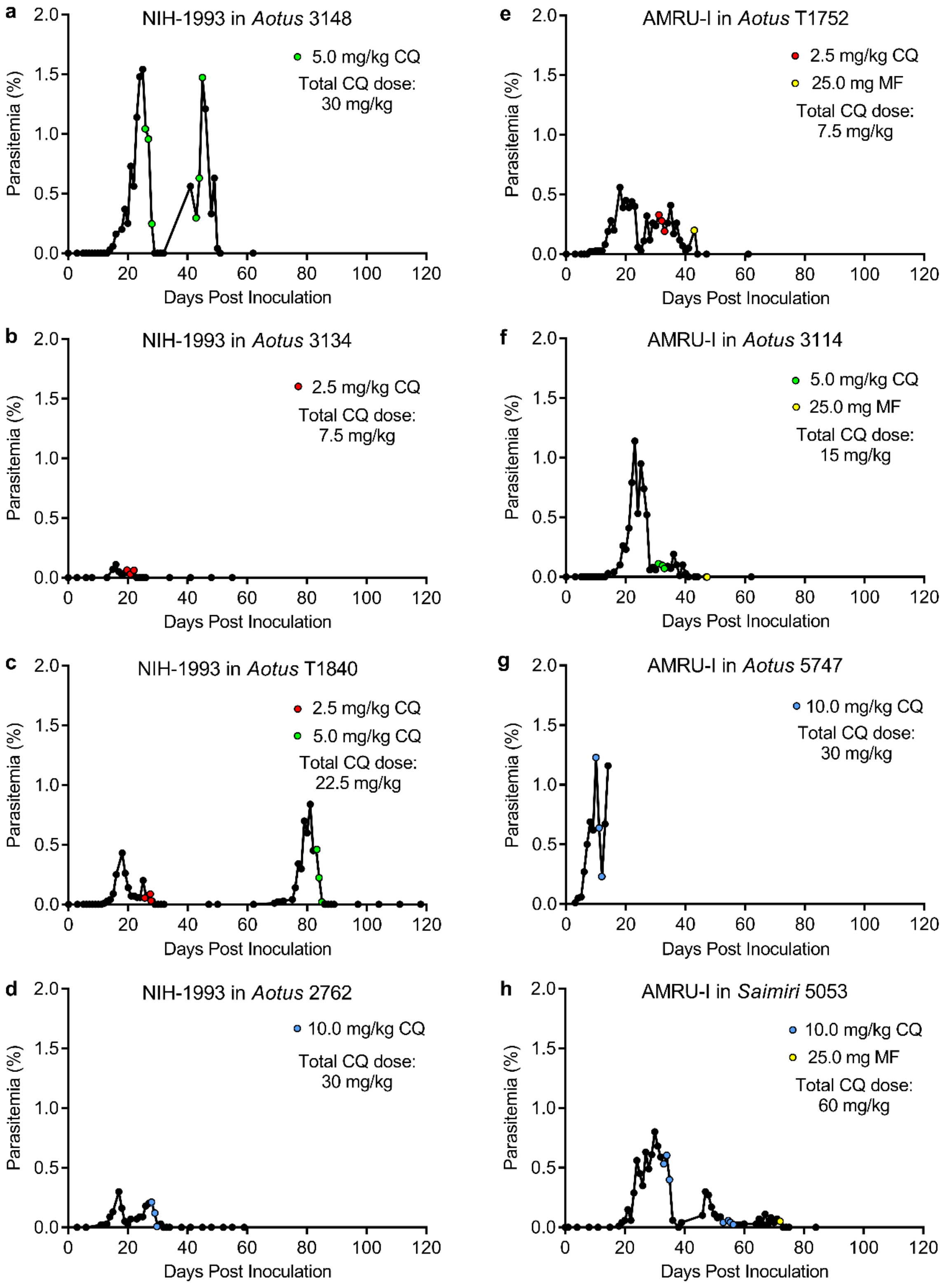

Supplementary Figure 1. Representative *P. vivax* NIH-1993 and AMRU-I responses to CQ treatments in *Aotus* or *Saimiri* monkeys. (a) *Aotus* 3148 inoculated with NIH-1993 shows parasite recrudescence when treated with 3 daily doses of 5 mg/kg CQ. A blood sample from 3148 prior to the first CQ treatment provided the NIH-1993 parental parasites for the genetic cross in the chimpanzee. **(b)** *Aotus* 3134 inoculated with NIH-1993 shows no parasite recrudescence after receiving 3 daily doses of 2.5 mg/kg CQ. **(c)** *Aotus* T1840 inoculated with NIH-1993 shows parasite recrudescence when treated with 3 daily doses of 2.5 mg/kg CQ, but not after an additional 3 daily doses of 5 mg/kg CQ. (**d**) *Aotus* 2762 inoculated with NIH-1993 shows no parasite recrudescence after receiving 3 daily doses of 10 mg/kg CQ. **(e)** *Aotus* T1752 inoculated with AMRU-I shows parasite recrudescence when treated with 3 daily doses of 2.5 mg/kg CQ. A blood sample from T1752 prior to the CQ treatment was co-inoculated with the NIH-1993 sample from 3148 into the chimpanzee. **(f)** *Aotus* 3114 inoculated with AMRU-I parasites shows resistance to 3 daily doses of 5 mg/kg. (**g**) *Aotus* 5747 inoculated with AMRU-I parasites confirms parasite resistance to 3 daily doses of 10 mg/kg. **(h)** In S*aimiri* 5053 AMRU-I parasites recrudesced after each of two consecutive treatments of 3 daily doses of 10 mg/kg CQ (total of 60 mg/kg). Parasitemia values are listed in **Supplementary Table 2**.

Supplementary Figure 2

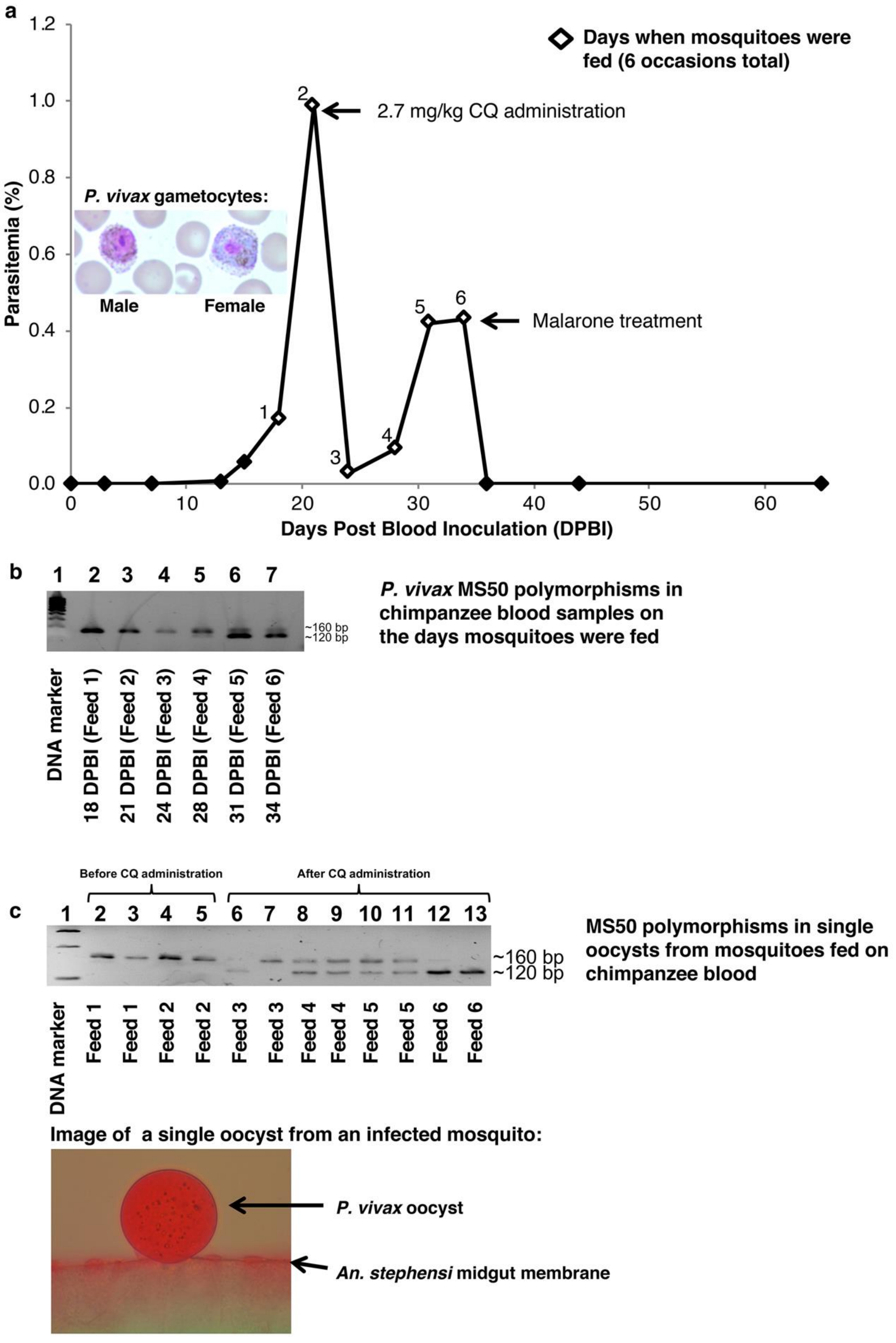

Supplementary Figure 2. Chimpanzee *P. vivax* parental lines infection and cross-fertilization in *Anopheles* mosquitoes. (a) Plot of parasitemia in the chimpanzee after inoculation with intraerythrocytic stages of *P. vivax* NIH-1993 from *Aotus* 3148 (**Supplementary Fig. 1a**) plus AMRU-I from *Aotus* T1752 (Supplementary Fig. 1e). Once gametocytes were observed in thin blood smears, *Anopheles* mosquitoes were fed on six occasions (points 1 – 6 in the graph) and blood samples were simultaneously taken to test for microsatellite alleles from parental lines. A single 2.7 mg/kg CQ dose was administered after mosquito feeding 2 to increase the numbers of CQ-resistant parental parasites without killing CQ-sensitive (CQ-S) parasites. Malarone treatment was administered for three days (arrow indicates day of first dose) to the chimpanzee after mosquito feeding 6 to cure the infection. Parasitemia values are provided in **Supplementary Table 3**. **(b)** *P. vivax* marker MS50 analysis shows the presence of a second DNA band in the chimpanzee blood at the time of feeds 4 – 6 (28, 31, and 34 DPBI), after administration of the 2.7 mg/kg CQ dose. (**c**) Results showing the presence of the two MS50 polymorphisms in single oocysts from mosquito batches of feeding 3, 4, and 5. An estimated size for each of the two observed microsatellite PCR products is indicated; Lane 1 shows 100 base pair reference standards. The micrograph shows an image of a single oocyst attached to an *An. stephensi* midgut.

Supplementary Figure 3

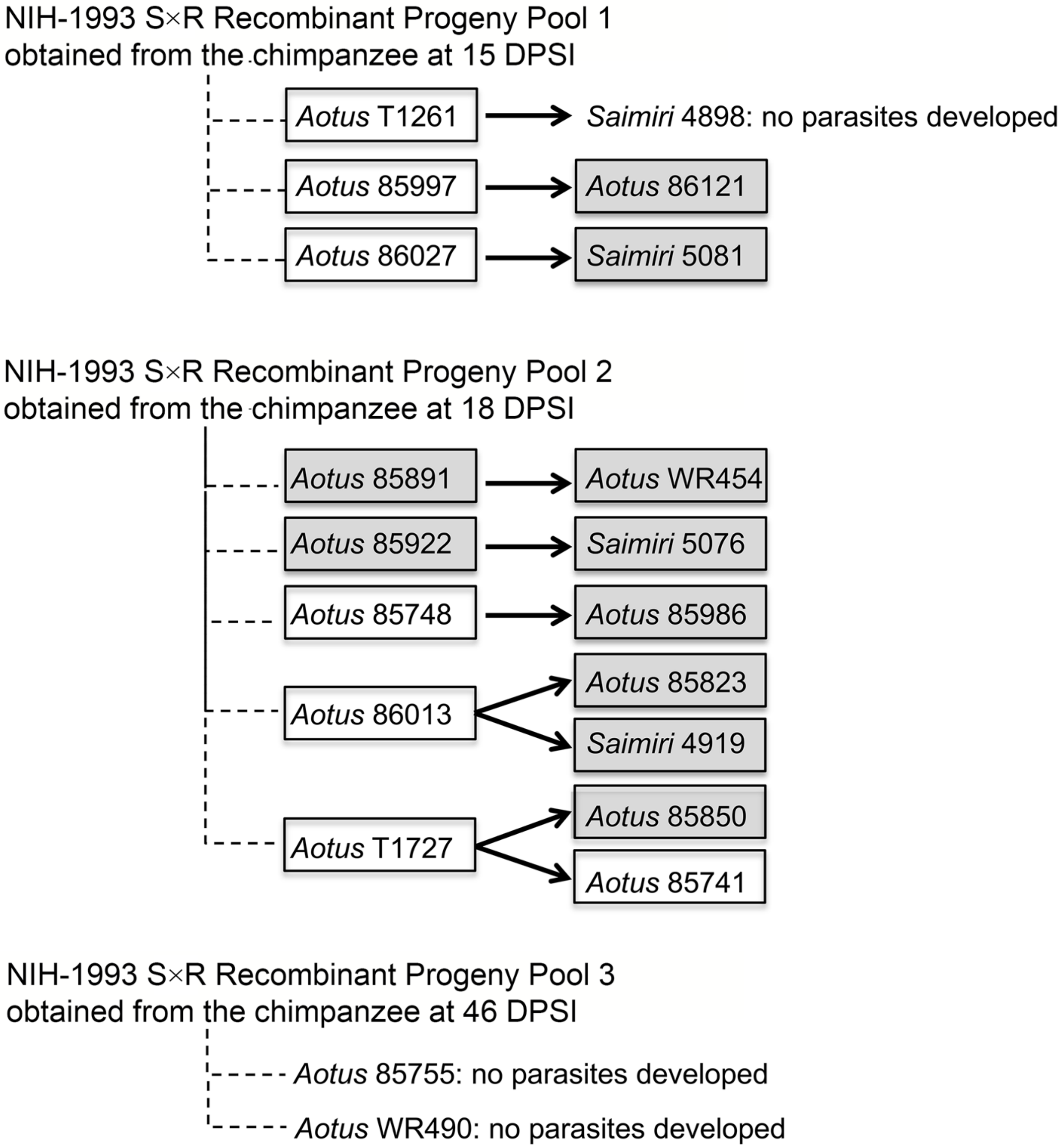

Supplementary Figure 3. Chart of *Aotus* and *Saimiri* monkey inoculations with NIH-1993 S×R progeny for pooled selection and linkage group analysis. Parasites from Progeny Pool 1 (PP1), collected from the chimpanzee at peak parasitemia (0.14%) on the 15th day post

sporozoite inoculation (DPSI), were used to infect *Aotus* T1261, 85997, and 86027. Parasites were observed in blood smears from these *Aotus* in 10 – 14 days. After collection and cryopreservation of blood samples, the PP1 infections were treated with 15 mg/kg total CQ (**Supplementary Table 8**); no recrudescences were observed in the 62-day follow-up period. Sub-inoculation of the *Aotus* T1261, 85997, and 86027 samples into three monkeys produced infections in *Aotus* 86121 and *Saimiri* 5081 but not in *Saimiri* 4898. These two successful infections were treated with 2 or 3 courses of CQ treatment (**Supplementary Fig. 4**), and recrudescent parasites were recovered from each animal for analysis and cryopreservation.

Parasites from chimpanzee Progeny Pool 2 (PP2) were inoculated into *Aotus* 85891, 85922, 85748, 86013 and T1727. All five monkeys developed parasitemias in 11 – 16 days. After collection and cryopreservation of blood samples, these PP2 infections were treated with 15 mg/kg total CQ (**Supplementary Table 8**) after which recrudescences occurred in *Aotus* 85891 and 85922 (**Supplementary Fig. 4**). Cryopreserved stocks from the PP2-infected monkeys were sub-inoculated as indicated into *Aotus* WR454, 85986, 85823, 85850, 85741, and *Saimiri* 5076 and 4919. Parasitemias in six of these sub-inoculated monkeys recrudesced after CQ total doses of 15 – 55 mg/kg (*Aotus* WR454, 85986, 85823, 85850, and *Saimiri* 5076 and 4919; **Supplementary Fig. 4; Supplementary Table 8).**

Ten of the 17 monkey infections provided recrudescent PP1 or PP2 populations for linkage group selection analysis (highlighted in gray). Inoculation of chimpanzee Progeny Pool 3 (PP3) samples into two *Aotus* did not produce a parasitemia or PCR signal of infection in either monkey.

Supplementary Figure 4

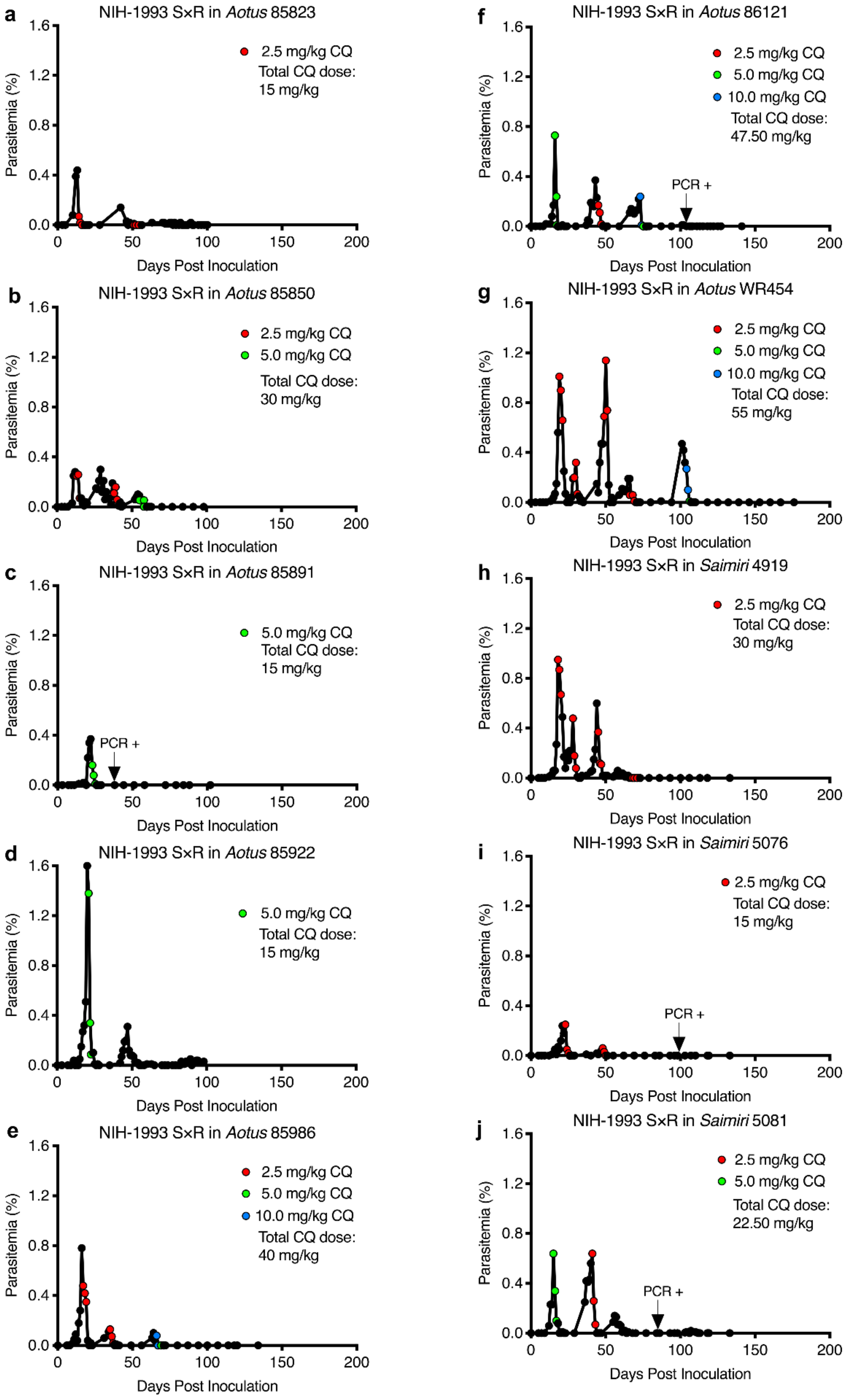

Supplementary Figure 4. Recrudescences after CQ treatment of *Aotus* and *Saimiri* monkeys infected with NIH-1993 S×R progeny pools PP1 or PP2. Each monkey received a total CQ dose of at least 15 mg/kg CQ. The parasitemia data for the graphs are provided in **Supplementary Table 7**. PCR + indicates detection by PCR but not by microscopy of blood smears.

Supplementary Figure 5

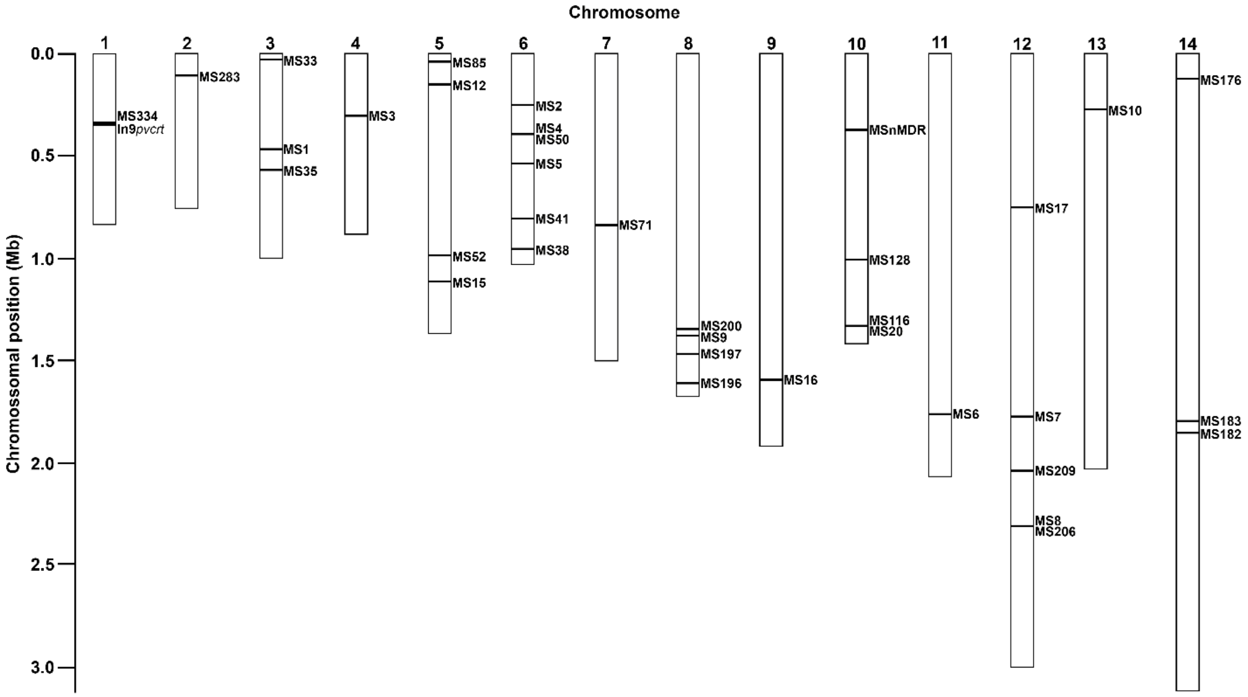

Supplementary Figure 5. Map showing chromosomal positions of the 37 microsatellite markers that distinguish parental polymorphisms in the NIH-1993 S×R cross.

Supplementary Figure 6

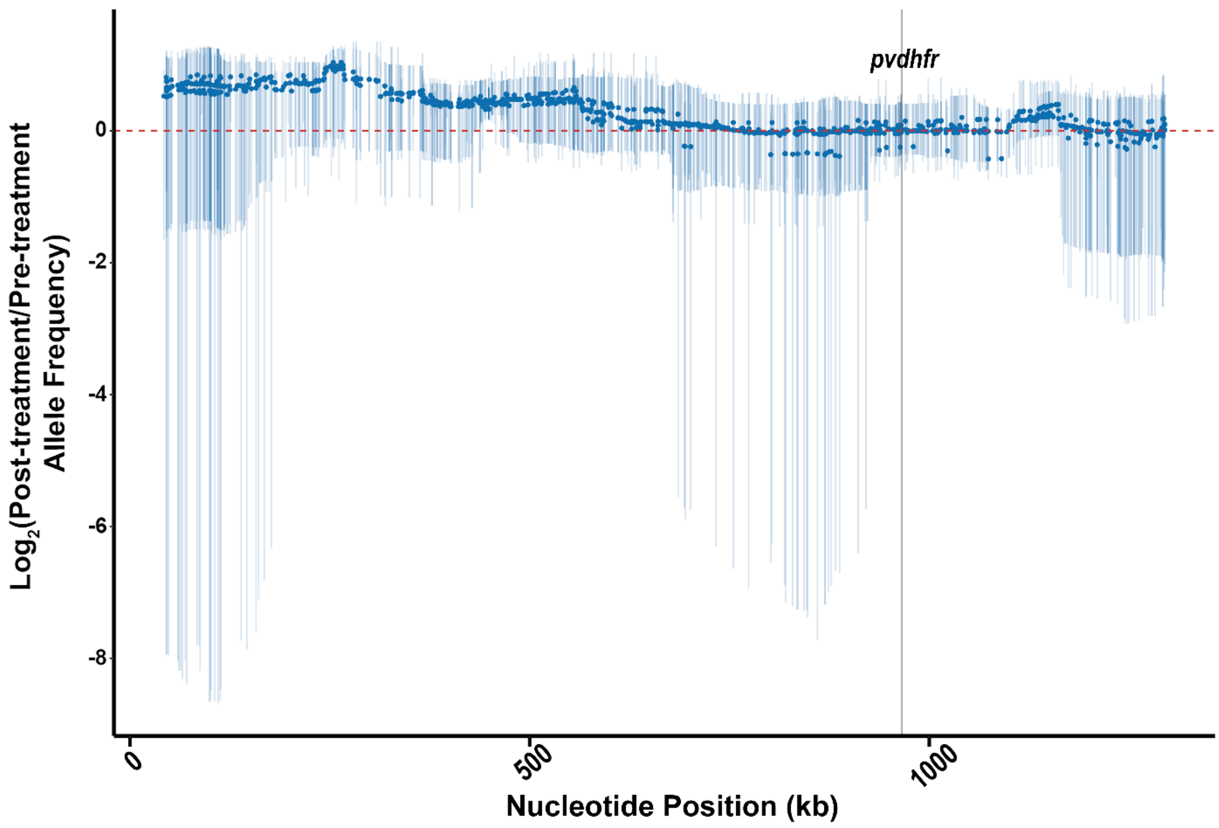

Supplementary Figure 6. Linkage group selection (LGS) analysis of chromosome 5 by targeted genomic sequencing. Aggregated Log_2-fold change of allele prevalence pre- and post-CQ treatment shown for LGS pairs from 6 of the monkeys (**Supplementary Figure 4;** *Aotus* 85823, 85850, 85922, 85986, WR454, and *Saimiri* 4919). Vertical blue lines are individual 95% bootstrap confidence intervals, and the red dotted line at 0 denotes no change from pre- to post-CQ treatment No apparent selection (was observed for the SNPs of the resistant parent in bootstrapped replicates. Position of the *P. vivax* dihydrofolate reductase gene (*pvdhfr*) is indicated. POS, nucleotide position in chromosome 5.

Supplementary Figure 7

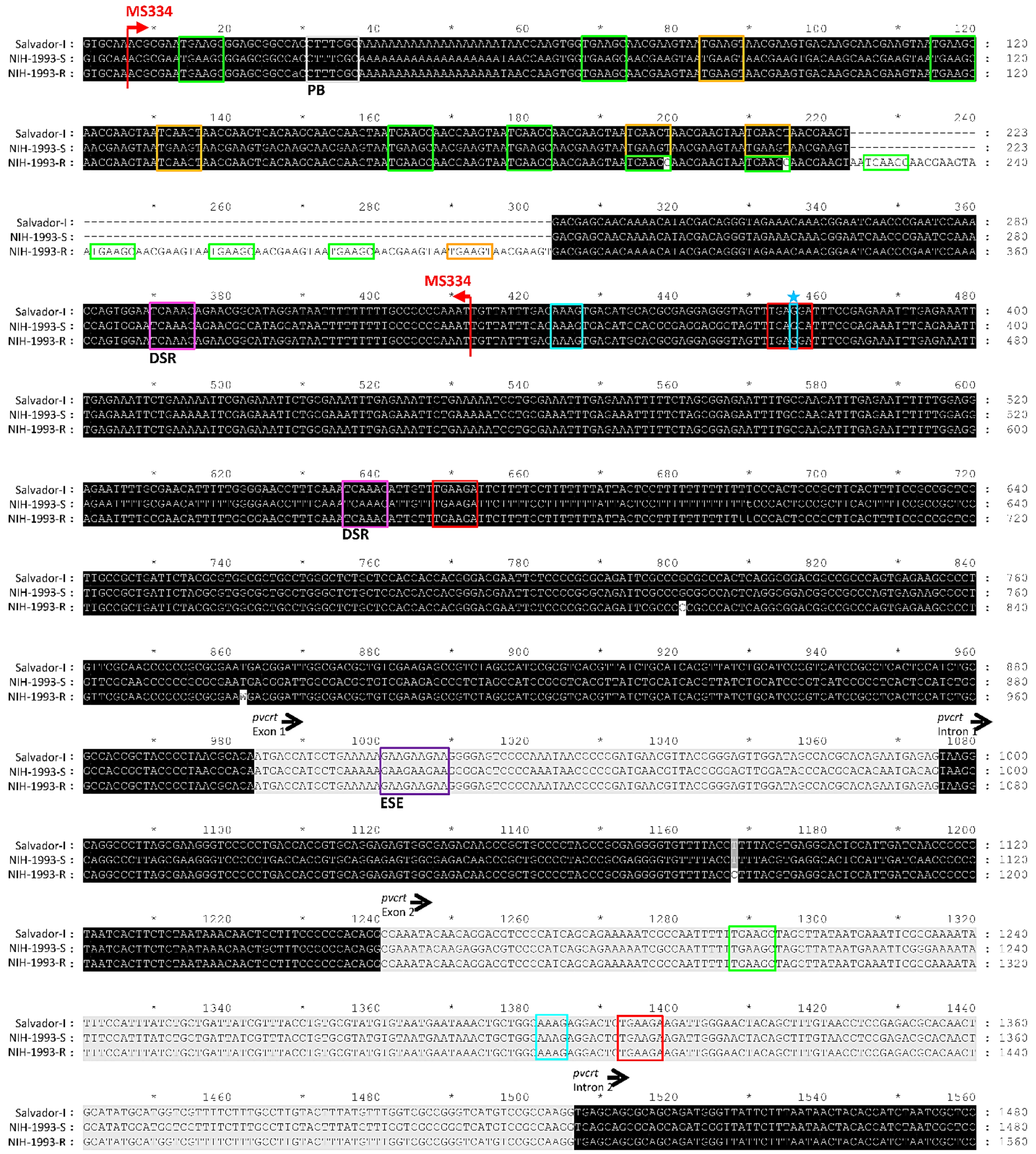

Supplementary Figure 7, continuation

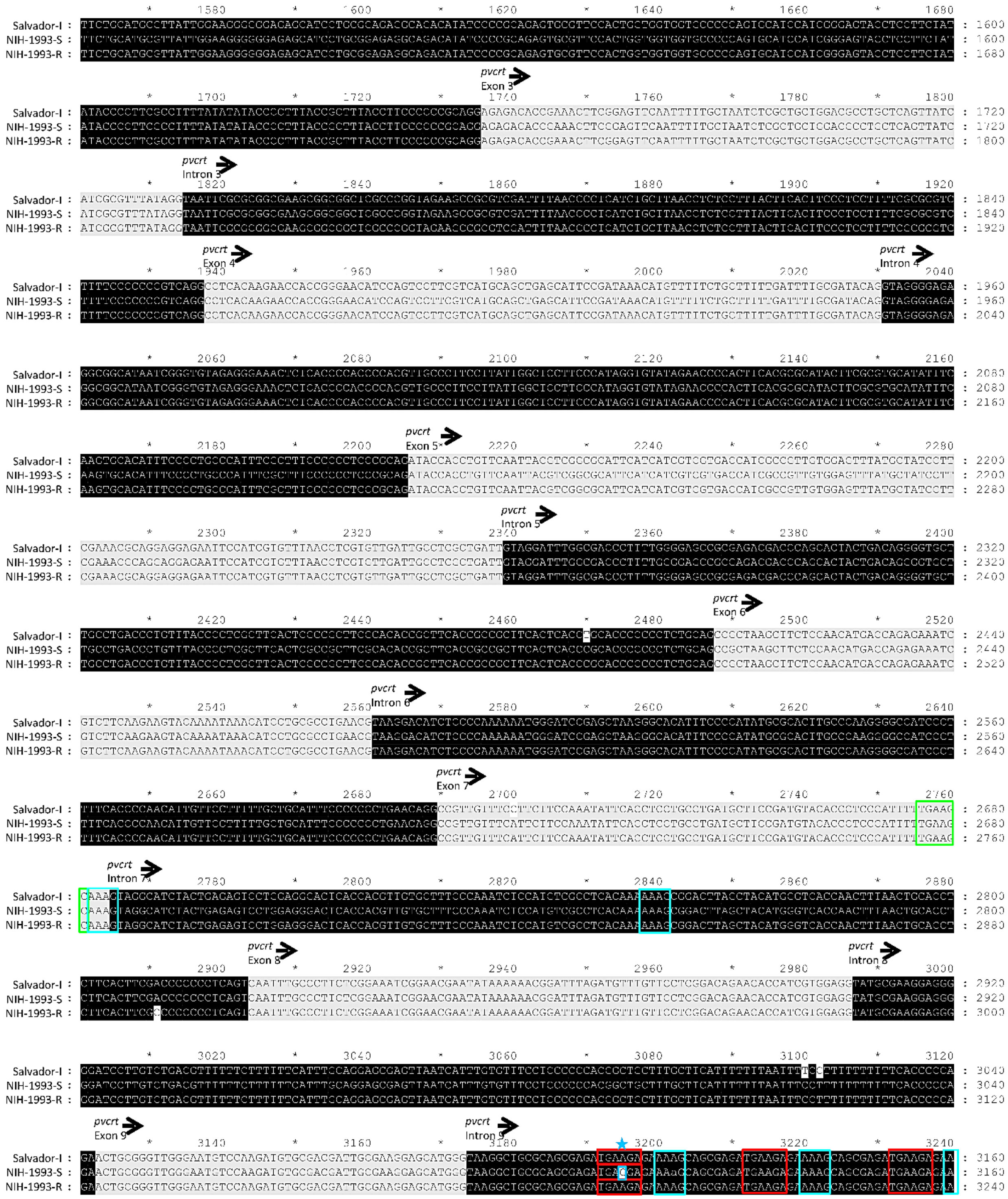

Supplementary Figure 7, continuation

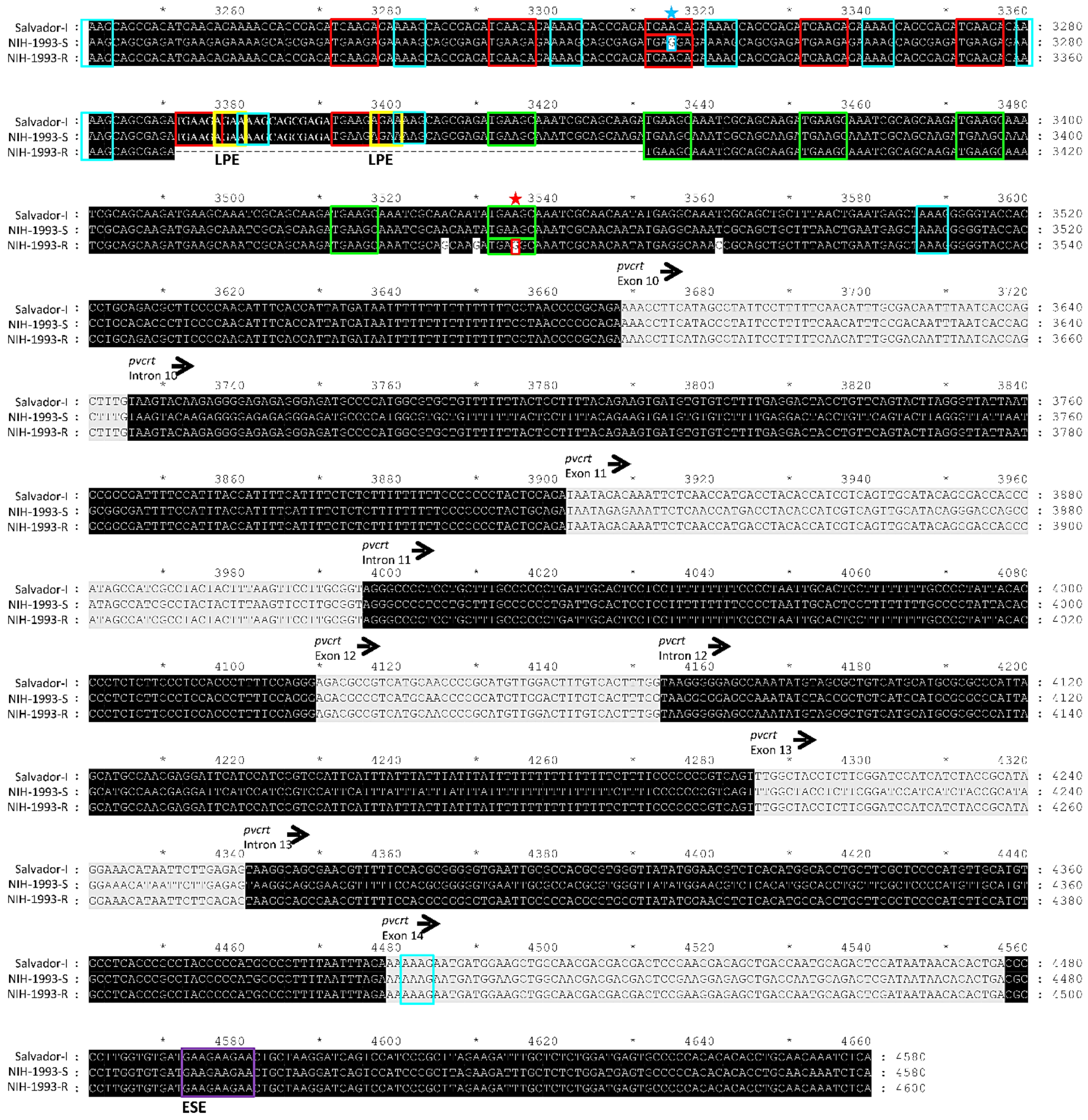

Supplementary Figure 7. Aligned sequences from the flanking regions, introns and exons of *pvcrt* in NIH-1993-S, NIH-1993-R and Salvador-I reference parasites. MS334 region is delimited by red lines with arrows. TGAAGH motifs found in the 5'-UTR, introns and exons are boxed in green (TGAAGC), orange (TGAAGT), and red (TGAAGA); versions of these repeats

containing a mutation disrupting the ‘GAAG’ core are shown with the substituting nucleotide boxed in blue and a star above. Resistant progeny selected by CQ contain an extra 82 bp, resulting in a longer 5'-UTR. Fewer repeats are present in intron 9 of NIH-1993-R than NIH-1993-S. PB (GCGAAAT, boxed in gray), Pausing Button element for RNA polymerase II found in *Drosophila*[1]; DSR (TCAAAC, boxed in pink), Determinant of Selective Removal motif that recruits RNA elimination machinery for cell cycle stage-specific gene silencing in yeast[2]; ESE (GAAGAAGAA, boxed in magenta), Exon Splicing Enhancer motif regulates splicing of human immunodeficiency virus type 1 through binding to serine arginine rich protein[3]; LPE (AGAAA, boxed in yellow), Late Pollen Element responsible for pollen time specific expression of tomato genes[4], also observed in promoter regions of *Arabidopsis* genes[5]; Dof (AAAG, boxed in light blue), core motifs recognized by members of the Dof transcription factor family, involved in light or pathogen induced gene expression in plants[6].

Supplementary Table 1. ***P. vivax*** **dihydrofolate reductase sequence polymorphisms in the NIH-1993 and Salvador-I lines**

***P. vivax* line**	***pvdhfr* nucleotide position & codon sequence**		
	**169**	**295**	**517**
PVX_089950 (Salvador-I)[7]	TTC	CAC	ATT
NIH-1993	CTC	AGC	TTT
***P. vivax* line**	**PvDHFR amino acid residue**		
	**57**	**99**	**173**
PVX_089950 (Salvador-I)[7]	F	H	I
NIH-1993	L	S	F

Amplification, cloning and sequence of NIH-1993 parental line gDNA showed a single *pvdhfr* allele. This distinguishes from the allele of the reference line Salvador-I by three amino acid substitutions: mutation of nucleotide 169 (T→C) results in codon 57 change from phenylalanine (F) to leucine (L); mutation of nucleotide 295 (C→A) results in codon 99 change from histidine (H) to a serine (S); and mutation of nucleotide 517 (A→T) results in codon 173 change from isoleucine (I) to F.

Supplementary Table 2. Parasitemia (%) development of NIH-1993 and AMRU-I infections in *Aotus* and *Saimiri* monkeys*

DPBI	NIH-1993				AMRU-I			
	Aotus 3148	*Aotus* 3134	*Aotus* T1840	*Aotus* 2762	*Aotus* T1752	*Aotus* 3114	*Aotus* 5747	*Saimiri* 5053
**0**	0	0	0	0	0	0		0
**1**								0
**3**	0	0	0	0	0	0	0.01	
**4**							0.05	0
**5**	0		0		0	0	0.06	
**6**	0	0	0	0	0	0	0.27	
**7**	0		0		0.001	0	0.50	
**8**	0	0	0		0.02	0	0.69	0
**9**	0		0		0.02	0.001	0.62	
**10**	0		0		0.03	0	1.23	
**11**	0.001		0.001	0.02	0.03	0.001	0.64	0
**12**	0.001		0.01	0.02	0.03	0.001	0.23	
**13**	0.001	0	0.03	0.03	0.08	0.001	0.67	
**14**	0.03		0.04	0.09	0.19	0.03	1.16	
**15**	0.06	0.07	0.09	0.13	0.28			0
**16**	0.16	0.11	0.25		0.20	0.04		
**17**		0.05		0.30				
**18**	0.20	0.03	0.43	0.16	0.56	0.10		0.01
**19**	0.37	0.04	0.26	0.05	0.39	0.26		0.04
**20**	0.25	0.06	0.14	0.03	0.45	0.23		0.06
**21**	0.73	0.03	0.07	0.07	0.39	0.41		0.15
**22**	0.56	0.06	0.07		0.44	0.79		0.06
**23**	1.14	0	0.06	0.07	0.40	1.14		0.29
**24**	1.48	0	0.06	0.09	0.06	0.53		0.56
**25**	1.54	0	0.20	0.09	0.03	0.95		0.45
**26**	1.04	0	0.06	0.18	0.11	0.74		0.35
**27**	0.96		0.08	0.20	0.32	0.52		0.63
**28**	0.25		0.03	0.21	0.12	0.06		0.49
**29**	0		0	0.12	0.26	0.08		0.61
**30**	0		0	0.01	0.24	0.06		0.80
**31**	0		0	0.03	0.33	0.11		0.68
**32**	0		0	0	0.28	0.10		0.59
**33**				0	0.19	0.07		0.53
**34**		0		0	0.26	0.09		0.60
**35**					0.41	0.07		0.40

DPBI	NIH-1993				AMRU-I			
	***Aotus* 3148**	***Aotus* 3134**	***Aotus* T1840**	***Aotus* 2762**	***Aotus* T1752**	***Aotus* 3114**	***Aotus* 5747**	***Saimiri* 5053**
**36**					0.17	0.19		0.06
**37**					0.26	0.10		
**38**				0	0.12	0.01		0.00
**39**					0.07	0.10		0.04
**40**					0.03	0.30		
**41**	0.56	0		0	0.05	0		
**43**	0.30				0.20	0		
**44**	0.63				0	0		
**45**	1.47			0	0			
**46**	1.21							0.10
**47**			0		0	0		0.30
**48**	0.33	0		0				0.27
**49**	0.63							0.17
**50**	0.04		0					0.11
**51**	0.00							0.08
**52**				0				0.09
**53**								0.04
**55**		0		0				0.05
**56**								0.03
**57**								0.02
**58**								0.02
**59**				0				0.02
**60**								0.03
**62**	0		0		0	0		
**64**								0.03
**65**								0.07
**66**								0.03
**67**								0.11
**68**								0.03
**69**			0.01					0.08
**70**			0.02					0.04
**71**			0.02					0.07
**72**			0.03					0.05
**73**								0
**74**								0
**75**			0.04					0
**76**			0.14					
**77**			0.34					
**78**			0.30					

DPBI	NIH-1993			AMRU-I				
	***Aotus* 3148**	***Aotus* 3134**	***Aotus* T1840**	***Aotus* 2762**	***Aotus* T1752**	***Aotus* 3114**	***Aotus* 5747**	***Saimiri* 5053**
**79**			0.70					
**80**			0.60					
**81**			0.84					
**82**			0.45					
**83**			0.46					
**84**			0.22					
**85**			0.02					
**86**			0					
**87**			0					
**88**			0					
**89**			0					
**97**			0					
**104**			0					
**111**			0					
**118**			0					

*These parasitemia values of *P. vivax* NIH-1993 and AMRU-I in *Aotus* and *Saimiri* are graphically displayed in **Supplementary Fig. 1**. Counts are from an estimated 10,000 erythrocytes in thin blood films fixed with methanol and stained with 10% Giemsa for 15 minutes. DPBI, days post blood inoculation. Cells are highlighted according to the antimalarial and the dose given on that day: red, 2.5 mg/kg CQ; green, 5 mg/kg CQ; blue, 10 mg/kg CQ; yellow, 25 mg single dose MF.

Supplementary Table 3. Development of *P. vivax* chimpanzee infection after blood-stage parasite inoculation

DPBI	Parasitemia (%)	PCR analysis	Procedures
0	0	-	IV inoculation with $5x10^6$ pRBC from NIH-1993 + $5x10^6$ pRBC from AMRU-I
3	0	-	Blood smear
7	0	-	Blood smear
13	0.01	-	Blood smear
15	0.06	Only NIH-1993-S parent detected	Blood smear; blood draw
18	0.17	Only NIH-1993-S parent detected	Blood smear; blood draw; *Anopheles* mosquito feeding 1
21	0.99	Only NIH-1993-S parent detected	Blood smear; blood draw; *Anopheles* mosquito feeding 2; 2.7 mg/kg CQ treatment after PCR detected only CQ-S alleles
24	0.03	Both NIH-1993-S and NIH-1993-R parents detected	Blood smear; blood draw; *Anopheles* mosquito feeding 3
28	0.09	Both parents detected	Blood smear; blood draw; *Anopheles* mosquito feeding 4
31	0.42	Both parents detected	Blood smear; blood draw; *Anopheles* mosquito feeding 5
34	0.43	Both parents detected	Blood smear, blood draw; *Anopheles* mosquito feeding 6; Malarone treatment
35	-	-	Malarone treatment
36	0	-	Blood smear; Malarone treatment
44	0	*Pv* negative by PCR	Blood smear; blood draw
65	0	*Pv* negative by PCR	Blood smear; blood draw

Parasitemia values represent the percentage of infected cells from counts of 10,000 erythrocytes in thin blood smears fixed with methanol and stained with 10% Giemsa for 15 minutes. DPBI, days post blood inoculation.

Supplementary Table 4. Genotypes of *P. vivax* lines inoculated into a chimpanzee

Microsatellite		Allele sizes in inoculum (bp)		Alleles detected in chimpanzee blood (bp)		
				Before low dose CQ	After low dose CQ	
**Name**	**Chr**	**Salvador-I**	**AMRU-I**		**NIH-1993-S**	**NIH-1993-R**
MS334	1	358	354	358	358	436
MS50	6	158	123	158	158	123
MS38	6	213	391	213	213	200
MS206	12	160	196	160	160	229

Chr, chromosome number; CQ, chloroquine; bp, base pairs. Samples were genotyped and analyzed blindly.

Supplementary Table 5. ***P. vivax*** **infections of** ***Anopheles*** **mosquitoes fed on chimpanzee blood**

DPSI	Blood-stage parasitemia	Mosquito species	Total fed	Mosquitoes infected	Average oocysts/ midgut	Average sporozoites/ mosquito	Viability of fresh sporozoites	Viability of cryopreserved sporozoites
18	0.17%	*An. stephensi*<br>*An. dirus*<br>*An. gambiae*<br>*An. freeborni*	5,000	60%	93	27,806	97% (Combined mosquitoes fed on 18 and 21 DPSI)	85% (Combined mosquitoes fed on 18 and 21 DPSI)
21	0.99%	*An. stephensi*<br>*An. dirus*<br>*An. gambiae*<br>*An. freeborni*	5,000	33%	135	12,195		
24	0.03%[*]	*An. stephensi*<br>*An. dirus*<br>*An. gambiae*<br>*An. freeborni*	3,700	-	-	-	-	-
28	0.09%	*An. stephensi*<br>*An. freeborni*	3,000	53%	9	7,797	94%	86%
31	0.42%	*An. stephensi*<br>*An. freeborni*	3,000	69%	142	34,957	96%	87%
34	0.43%	*An. stephensi*<br>*An. freeborni*	3,600	88%	85	42,070	94%	90%

[*]Parasites from this post-CQ feeding infected mosquitoes were at levels too low to obtain sporozoites for cryopreservation.

Viability of fresh and cryopreserved sporozoites were determined in hepatocyte invasion assays as described[8].

DPSI, days post sporozoite inoculation.

Supplementary Table 6. Development of *P. vivax* in chimpanzee after NIH-1993 S×R recombinant sporozoite inoculation

DPSI	Parasitemia (%)	PCR analysis	Procedures	CQ (mg/kg)
0	0		IV inoculation of 10.5 million sporozoites (from mosquito feeds 4 and 5)	
8	<0.01	*Pv* positive	Blood smear; blood draw	
11	0.06		Blood smear; blood draw	
13	0.14		Blood smear; blood draw	
15	0.14		Blood smear; blood draw (PP1); sub-inoculation into *Aotus*	
18	0.005		Blood smear; blood draw (PP2); sub-inoculation into *Aotus*; CQ treatment	5.0
19			CQ treatment	5.0
20	0	*Pv* positive	Blood smear; blood draw; CQ treatment	5.0
21	0	*Pv* positive	Blood smear; blood draw	
28	0	*Pv* positive	Blood smear; blood draw	
32	0	*Pv* positive	Blood smear; blood draw	
39	<0.01		Blood smear; blood draw	
42	0.01		Blood smear; blood draw	
43			Blood smear; blood draw (PP3); sub-inoculation into *Aotus*	
50	0	*Pv* positive	Blood smear; blood draw	
53	0	*Pv* positive	Blood smear; blood draw; CQ treatment	2.5
54			CQ treatment	2.5
55			CQ treatment	2.5
56	0	*Pv* positive	Blood smear; blood draw	
63	0	*Pv* positive	Blood smear, blood draw	
71	0	*Pv* positive	Blood smear; blood draw	
78	0	*Pv* positive	Blood smear, blood draw	
84	0	*Pv* positive	Blood smear; blood draw	
92	0	*Pv* positive	Blood smear, blood draw	
102	0	*Pv* positive	Malarone and Primaquine treatment	
103			Malarone and Primaquine treatment	
104			Malarone and Primaquine treatment	
118	0	*Pv* negative	Blood smear; blood draw	

DPSI, days post sporozoite inoculation; *Pv*, *P. vivax*; CQ, chloroquine; PP1, PP2, and PP3, progeny pools 1, 2, and 3, respectively.

Supplementary Table 7. Parasitemia (%) development of NIH-1993 S×R progeny in *Aotus* and *Saimiri* monkeys*

DPBI	*Aotus* 85823	*Aotus* 85850	*Aotus* 85891	*Aotus* 85986	*Aotus* 85922	*Aotus* 86121	*Aotus* WR454	*Saimiri* 4919	*Saimiri* 5076	*Saimiri* 5081
0	0	0	0	0	0	0	0	0	0	0
3	0	0	0		0	0	0			
5	0	0						0	0	0
6				0		0	0			
7		0	0	0	0			0	0	0
8				0		0	0			
9			0		0			0	0	0
10	0.08	0.03		0.02		0.02	0			
11		0.25	0	0.05	0.04					
12	0.39	0.28		0.09	0			0.01	0	0.06
13	0.44	0.25		0.04	0.02	0.02	0.01	0.02		0.23
14	0.07	0.26	0.01	0.18	0	0.08	0.02	0.03	0.01	0.23
15	0.02	0.07		0.28	0.05	0.17	0.04	0.06		0.64
16	0	0.07	0.01	0.78	0.15	0.73	0.07	0.06	0.05	0.34
17	0	0.03	0.02	0.48	0.27	0.24	0.15	0.27	0.01	0.10
18	0	0.01	0.02	0.42	0.32	0	0.56	0.95	0.07	0.08
19	0	0.04	0	0.35	0.51	0	1.01	0.87	0.05	0
20	0	0.03	0.22	0.04	1.60	0	0.90	0.67	0.12	0
21	0		0.34	0	1.38	0.01	0.66	0.49	0.24	0.01
22			0.37	0.02	0.34	0	0.25	0.17	0.18	0
23			0.16	0	0.09	0	0.07	0.08	0.25	0
24			0.08		0.10	0	0	0.20	0.05	
25			0.01		0.01		0.04	0.14	0.02	
26		0.15	0		0		0	0.22	0	
27		0.14	0		0.01		0.04	0.19	0	

**DPBI**	***Aotus* 85823**	***Aotus* 85850**	***Aotus* 85891**	***Aotus* 85986**	***Aotus* 85922**	***Aotus* 86121**	***Aotus* WR454**	***Saimiri* 4919**	***Saimiri* 5076**	***Saimiri* 5081**
28	0	0.21	0		0		0.19	0.48	0	
29		0.30	0				0.20	0.18	0	0
30		0.12				0	0.32	0.08		
31		0.21		0.06			0.07	0.01		
32		0.06					0.05	0		
33		0.12					0.01	0		
34		0.07		0.11			0.02	0.02		
35		0.07		0.13	0		0			
36		0.02		0.07						0.25
37		0.19		0.02		0.01		0.02	0.01	0.42
38		0.11	0	0		0.05				0.41
39		0.16		0.01		0.04				0.43
40		0.06		0		0.19		0.05	0	0.56
41		0.01		0		0.17		0.06		0.64
42	0.14	0.04			0.02	0.16		0.15		0.26
43		0			0.07	0.37		0.23		0.07
44		0.01	0		0.12	0.23	0.15	0.60	0.02	0
45					0.19	0.17	0.08	0.37	0.01	0
46	0.03				0.2	0.11	0.32	0.12	0.02	0
47	0				0.31	0.02	0.47	0.11	0.04	0
48	0.02				0.12	0	0.47	0.02	0.06	
49	0.01			0	0.08	0	0.69	0.01	0.03	
50	0				0.08	0	1.14	0.02	0	
51	0.01		0		0.07	0	0.74	0.02	0	
52	0	0.06			0.03		0.14			
53	0	0.08			0.02		0			
54	0	0.10			0.02		0.04			
55	0	0.09			0		0	0.01	0	0.09

DPBI	*Aotus* 85823	*Aotus* 85850	*Aotus* 85891	*Aotus* 85986	*Aotus* 85922	*Aotus* 86121	*Aotus* WR454	*Saimiri* 4919	*Saimiri* 5076	*Saimiri* 5081
56	0	0.05		0	0			0.02		0.14
57		0.05						0.03		0.13
58		0	0		0			0.06		0.07
59		0				0	0.06	0.02		0.07
60		0			0.01			0.02		0.06
61		0					0.11	0.04		0.01
62		0					0.09	0.03	0	0.02
63	0.02	0		0.06	0.01		0.09	0.01		0.02
64				0.1			0.1	0.01		0
65				0.04	0		0.19	0.02		0.01
66				0.08		0.11	0.19	0		
67				0		0.14	0.06	0		
68				0		0.12	0.06	0		0
69				0		0.1	0.01	0	0	
70	0.02	0		0	0	0.13	0	0		0
71	0.02			0		0.13	0	0		
72	0.01		0	0	0	0.22	0	0		
73	0.01					0.24	0			
74	0.01				0	0.01				
75	0.02					0				
76	0.02					0			0	
77	0	0			0	0				0
78	0.02					0				
79	0		0		0	0		0		
80	0.01			0			0			
81	0				0					
82	0.02				0					
83	0				0.03				0	

DPBI	*Aotus* 85823	*Aotus* 85850	*Aotus* 85891	*Aotus* 85986	*Aotus* 85922	*Aotus* 86121	*Aotus* WR454	*Saimiri* 4919	*Saimiri* 5076	*Saimiri* 5081
84	0	0	0		0.01					0
85	0				0.01					
86	0				0.03			0	0	0
87	0			0	0.02	0	0.01			
88	0.01		0		0.02					
89	0.02				0.05					
90	0				0					
91	0	0			0.02					
92					0.02					
93					0.04			0	0	0
94	0			0	0.04	0	0			
95					0.03					
96	0								0	0
98	0	0			0.03				0	0
100	0			0				0		
101						0.01	0.47			
102			0			0.01	0.42			
103							0.32		0	0.005
104						0	0.27			0.01
105							0.10			0.01
106						0	0.01	0		0
107				0		0	0		0	0.02
108						0	0			
109							0			
110							0		0	0.01
111						0				0.005
112										0
113						0		0		0
114				0						

DPBI	*Aotus* 85823	*Aotus* 85850	*Aotus* 85891	*Aotus* 85986	*Aotus* 85922	*Aotus* 86121	*Aotus* WR454	*Saimiri* 4919	*Saimiri* 5076	*Saimiri* 5081
115						0				
118				0		0	0	0	0	0
119									0	0
120				0		0				
122						0				
125						0	0			
127						0				
132							0			
133								0	0	0
134				0						
139							0			
141						0				
146							0			
153							0			
160							0			
167							0			
176							0			

*Values are graphically displayed in **Supplementary Fig. 4**. DPBI, days post blood inoculation. Cells are highlighted according to the antimalarial drug and dose given on indicated day: red, 2.5 mg/kg CQ; green, 5 mg/kg CQ; blue, 10 mg/kg CQ.

Supplementary Table 8. Chloroquine plasma concentrations in *Aotus* and *Saimiri*

Species	Animal ID	Treatment Initiation (DPBI)	Plasma Collection (DPBI)	CQ dose	CQ (ng/ml)	Desethyl-CQ (ng/ml)	Didesethyl-CQ (ng/ml)
Aotus	85823	14	17	7.5 mg/kg	49.02	DUL	-
		49	52	7.5 mg/kg (2nd CQ administration)	31.80	DUL	+
Aotus	85850	14	17	7.5 mg/kg	37.34	DUL	+
		38	41	7.5 mg/kg (2nd CQ administration)	82.57	5.36	+
		56	59	15.0 mg/kg (3rd CQ administration)	59.44	5.12	-
Aotus	85891	23	26	15.0 mg/kg	28.60	DUL	-
Aotus	85922	21	24	15.0 mg/kg	160.40	122.3	+
Aotus	85986	17	20	7.5 mg/kg	41.96	DUL	+
		35	38	7.5 mg/kg (2nd CQ administration)	45.12	DUL	+
		68	71	25.0 mg/kg (3rd CQ administration)	32.69	20.63	+
Aotus	86121	16	19	15.0 mg/kg	21.80	DUL	+
		45	48	7.5 mg/kg (2nd CQ administration)	17.90	DUL	+
		75	78	25.0 mg/kg (3rd CQ administration)	18.99	15.5	+
Aotus	WR454	19	22	7.5 mg/kg	38.10	18.90	+
		29	32	7.5 mg/kg (2nd CQ administration)	38.98	7.20	+
		49	52	7.5 mg/kg (3rd CQ administration)	20.50	DUL	+
		67	70	7.5 mg/kg (4th CQ administration)	67.10	110.20	+
		104	107	25.0 mg/kg (5th CQ administration)	46.50	20.70	+
Saimiri	4919	18	21	7.5 mg/kg	63.60	ND	+
		28	31	7.5 mg/kg (2nd CQ administration)	47.50	ND	+
		45	48	7.5 mg/kg (3rd CQ administration)	26.20	ND	+
		66	69	7.5 mg/kg (4th CQ administration)	36.70	33.40	+
Saimiri	5076	23	26	7.5 mg/kg	5.10	ND	+
		48	51	7.5 mg/kg (2nd CQ administration)	DUL	ND	+
Saimiri	5081	15	18	15.0 mg/kg	ND	ND	-
		41	44	7.5 mg/kg (2nd CQ administration)	28.20	DUL	+

Post-treatment plasma concentrations of CQ and metabolites (desethyl-CQ, and didesethyl-CQ) in monkeys infected with NIH-1993 S×R progeny.

ID, animal identification number; DPBI, days post blood inoculation; CQ, chloroquine; DUL, detected under the limit of quantification; ND, not detected. “Treatment Initiation” refers to the first day CQ was given for the corresponding dose treatment administration; “Plasma Collection” refers to the day blood was collected for plasma separation and CQ measurement.

Supplementary Table 9. Ratios of parental microsatellite intensities after and before CQ treatment, from CQ-treated monkeys infected with NIH-1993 S×R progeny

**Chromosome**	**Marker**	**Location (kb)**	***Aotus* 85823**	***Aotus* 85850**	***Aotus* 85891**	***Aotus* 85896**	***Aotus* 85922**	***Aotus* 86121**	***Aotus* WR454**	***Saimiri* 4919**	***Saimiri* 5076**	***Saimiri* 5081**
1	MS334	330.05	120.40	1.22	30.73	5.82	17.43	3.12	71.88	38.05	2.03	3.35
1	In9*pvcrt*	333.13	26.11	1.04	3.07	1.72	14.34	5.53	16.05	10.86	3.74	2.76
2	MS283	121.34	0.32	1.35	3.33	0.29	0.82	1.25	0.67	1.67	1.16	0.56
3	MS33	30.51	0.09	0.38	26.76	0.83	0.75	1.17	0.01	2.00	1.47	0.10
3	MS1	452.00	0.59	0.22	2.89	1.67	9.15	0.26	0.17	0.28	0.48	7.82
3	MS35	574.00	3.55	6.56	0.71	0.29	0.11	2.45	35.83	17.26	0.53	0.15
4	MS3	296.60	0.36	0.50	0.36	0.14	0.07	0.56	1.73	32.12	1.11	0.10
5	MS85	45.90	4.82	0.96	1.82	1.43	1.09	0.19	40.81	0.06	0.56	1.07
5	MS12	70.29	1.96	0.86	0.03	0.56	0.23	0.58	51.35	0.13	1.11	0.55
5	MS52	971.36	45.76	0.60	4.06	0.31	3.26	16.26	0.79	4.24	37.60	9.93
5	MS15	1110.66	5.69	0.62	14.25	4.45	4.25	8.97	1.68	0.95	1.84	25.99
6	MS2	256.39	2.38	2.60	1.55	3.12	6064.32	0.71	10.50	0.51	0.08	6.58
6	MS4	390.28	18.33	1.07	1.16	0.00	14.58	0.50	59.32	1.23	0.82	87.14
6	MS50	390.46	250.40	1.87	0.57	0.13	1.93	0.97	59.27	1.07	2.54	7.30
6	MS5	538.57	19.28	1.19	0.03	0.38	6.29	0.39	1.95	0.72	0.54	3.05
6	MS41	834.54	106.91	0.32	2140.06	0.08	0.04	0.20	0.34	40.01	0.42	0.15
6	MS38	957.22	541.68	3.18	2727.41	1.89	16.70	0.60	10.80	0.06	1.69	4.35
7	MS71	855.542	16.67	1.79	0.83	0.77	0.53	2.00	0.48	0.31	0.47	1.11
8	MS200	1358.29	0.02	1.51	651.15	0.14	10.10	1.28	0.83	0.08	0.53	2.20
8	MS9	1394.39	0.00	0.27	0.01	0.48	2.09	0.44	1.02	0.19	0.35	1.01
8	MS197	1469.90	0.00	1.31	0.18	1.25	6.95	1.01	0.64	3.50	1.12	10.23
8	MS196	1631.31	63.18	1.53	21.63	0.67	0.18	30.93	1.13	11.65	1.11	0.16
9	MS16	1593.22	154.96	0.78	0.85	0.36	9.00	0.32	0.18	1.41	0.48	2.23
10	MSnMDR	376.22	0.03	1.17	0.41	0.00	11.14	22.73	1.06	261.23	0.84	16.87
10	MS128	1012.30	0.18	2.87	129.73	0.83	0.03	0.32	0.42	75.49	0.59	5.00
10	MS116	1345.58	0.03	0.32	0.04	0.53	3729.75	1.76	0.19	0.03	0.32	4.85
10	MS20	1345.79	0.02	0.23	0.79	1.12	69.78	1.69	1.30	0.01	0.36	0.10
11	MS6	1760.92	0.52	0.45	0.23	0.34	8.94	2.12	69.07	4.89	0.62	1.75
12	MS17	753.02	0.95	0.61	1.21	0.91	0.96	0.84	0.80	1.36	1.12	1.36

**Chromosome**	**Marker**	**Location (Mb)**	***Aotus* 85823**	***Aotus* 85850**	***Aotus* 85891**	***Aotus* 85896**	***Aotus* 85922**	***Aotus* 86121**	***Aotus* WR454**	***Saimiri* 4919**	***Saimiri* 5076**	***Saimiri* 5081**
12	MS7	1185.29	0.03	0.51	0.68	0.30	2.42	1.81	5.18	3.01	0.43	0.39
12	MS209	2068.21	0.24	0.96	10.48	0.24	3.45	0.20	1.14	0.13	0.56	1.11
12	MS8	2322.34	0.99	1.55	0.00	3.33	0.37	4.89	0.29	13.51	1.61	0.93
12	MS206	2322.51	1.00	1.31	0.25	0.77	0.42	5.82	0.15	15.82	0.56	0.67
13	MS10	276.46	1.08	1.78	0.07	0.35	18.28	4.73	3.27	147.23	1.04	0.10
14	MS176	132.61	1.02	0.68	0.17	0.28	5.76	0.37	3.13	0.18	1.96	0.64
14	MS183	1777.62	13.84	1.25	103.77	1.67	2807.63	0.34	0.16	13.67	0.32	4.86
14	MS182	1848.55	0.55	1.24	1242.07	1.00	2626.60	0.74	0.15	16.04	0.54	0.20

Samples were genotyped and analyzed blindly.

Supplementary Table 10. ***P. vivax*** **single nucleotide polymorphisms in a CQ-selected 76 kb region of chromosome 1**

Chromosome	Position	Reference	Alternate
Pv_Sal1_chr01	292088	C	G
Pv_Sal1_chr01	315101	C	T
Pv_Sal1_chr01	317453	G	A
Pv_Sal1_chr01	317633	T	A
Pv_Sal1_chr01	321788	G	A
Pv_Sal1_chr01	323546	C	T
Pv_Sal1_chr01	323549	A	C
Pv_Sal1_chr01	323562	C	G
Pv_Sal1_chr01	324779	C	G
Pv_Sal1_chr01	326527	C	T
Pv_Sal1_chr01	328920	A	G
Pv_Sal1_chr01	332683	C	A
Pv_Sal1_chr01	332874	A	C
Pv_Sal1_chr01	337126	T	C
Pv_Sal1_chr01	337135	G	A
Pv_Sal1_chr01	338230	C	T
Pv_Sal1_chr01	339596	G	T
Pv_Sal1_chr01	341005	C	G
Pv_Sal1_chr01	342879	C	T
Pv_Sal1_chr01	344889	A	G
Pv_Sal1_chr01	346099	G	A
Pv_Sal1_chr01	347097	G	T
Pv_Sal1_chr01	350092	C	A
Pv_Sal1_chr01	350146	C	G
Pv_Sal1_chr01	350291	G	A
Pv_Sal1_chr01	350316	A	G
Pv_Sal1_chr01	351418	C	T
Pv_Sal1_chr01	352312	G	A
Pv_Sal1_chr01	352610	T	C
Pv_Sal1_chr01	354814	C	G
Pv_Sal1_chr01	355432	T	C
Pv_Sal1_chr01	360261	T	C
Pv_Sal1_chr01	361189	C	A
Pv_Sal1_chr01	362467	T	C
Pv_Sal1_chr01	363634	A	G

Pv_Sal1_chr01	363892	T	G
Pv_Sal1_chr01	363893	A	G
Pv_Sal1_chr01	365828	T	C
Pv_Sal1_chr01	365974	A	G
Pv_Sal1_chr01	366280	T	C
Pv_Sal1_chr01	367599	T	A
Pv_Sal1_chr01	368094	C	G

“Reference” refers to nucleotides of the *P. vivax* Salvador-I genome sequence and “Alternate” to the SNPs detected in the NIH-1993 samples.

Supplementary Table 11. Relative *pvcrt* transcript levels in *Aotus* and *Saimiri* infected with NIH-1993 S×R parasites

Sample	*pvseryl-tRNA synthetase* Ct	*pvcrt* Ct	ΔCt (*pvcrt* Ct - average *pvseryl-tRNA synthetase* Ct)	ΔΔCt (ΔCt - average ΔCt prior to CQ treatment)	$2^{-\Delta\Delta Ct}$ (*pvcrt* transcription relative to its average transcription prior to CQ treatment)
***Aotus* 85986**					
**Sample #1 prior to CQ treatment**	24.51	22.89	-1.86	-0.08	1.06
	24.98	23.05	-1.70	0.08	0.95
**Average ± SD**	24.74 ± 0.33	22.97 ± 0.11	-1.77 ± 0.11	0 ± 0.11	1 ± 0.08
**Sample #1 after CQ treatment**	28.14	25.77	-1.68	0.09	0.94
	26.76	25.23	-2.22	-0.45	1.36
**Average ± SD**	27.45 ± 0.98	25.50 ± 0.38	-1.95 ± 0.38	-0.18 ± 0.38	1.13 ± 0.30
**Sample #2 after CQ treatment**	25.75	21.47	-4.31	-2.53	5.78
	25.80	21.20	-4.58	-2.80	6.96
**Average ± SD**	25.77 ± 0.04	21.33 ± 0.19	-4.44 ± 0.19	-2.67 ± 0.19	6.34 ± 0.84
***Aotus* 86121**					
**Sample #1 prior to CQ treatment**	19.87	18.03	-1.85	-0.255	1.19
	19.9	18.54	-1.34	0.255	0.84
**Average ± SD**	19.88 ± 0.02	18.28 ± 0.36	-1.6 ± 0.36	0 ± 0.36	1 ± 0.25
**Sample #1 after CQ treatment**	23.3	19.69	-3.54	-1.94	3.83
	23.16	19.76	-3.47	-1.87	3.65
**Average ± SD**	23.23 ± 0.10	19.72 ± 0.05	-3.50 ± 0.05	-1.90 ± 0.05	3.74 ± 0.13
**Sample #2 after CQ treatment**	24.26	19.76	-4.82	-3.22	9.35
	24.91	20.20	-4.38	-2.78	6.89
**Average ± SD**	24.58 ± 0.46	19.98 ± 0.31	-4.605 ± 0.31	-3.00 ± 0.31	8.02 ± 1.74

Sample	*pvseryl-tRNA synthetase* Ct	*pvcrt* Ct	ΔCt (*pvcrt* Ct - average *pvseryl-tRNA synthetase* Ct)	ΔΔCt (ΔCt - average ΔCt prior to CQ treatment)	$2^{-\Delta\Delta Ct}$ (*pvcrt* transcription relative to its average transcription prior to CQ treatment)
***Aotus* WR454**					
**Sample #1 prior to CQ treatment**	21.06	20.26	-0.85	-0.07	1.05
	21.16	20.40	-0.71	0.07	0.95
**Average ± SD**	21.11 ± 0.07	20.33 ± 0.10	-0.78 ± 0.10	0 ± 0.10	1 ± 0.07
**Sample #1 after CQ treatment**	22.73	21.28	-1.495	-0.715	1.64
	22.82	21.14	-1.635	-0.855	1.81
**Average ± SD**	22.78 ± 0.06	21.21 ± 0.10	-1.565 ± 0.10	-0.78 ± 0.10	1.72 ± 0.11
***Saimiri* 4919**					
**Sample #1 prior to CQ treatment**	22.84	22.83	-0.185	-0.11	1.08
	23.19	23.05	0.035	0.11	0.92
**Average ± SD**	23.01 ± 0.25	22.94 ± 0.15	-0.07 ± 0.15	0 ± 0.15	1 ± 0.10
**Sample #1 after CQ treatment**	23.64	21.82	-1.73	-1.655	3.15
	23.46	22.09	-1.46	-1.385	2.61
**Average ± SD**	23.55 ± 0.13	21.95 ± 0.19	-1.59 ± 0.19	-1.52 ± 0.19	2.87 ± 0.38
***Saimiri* 5081**					
**Sample #1 prior to CQ treatment**	23.41	22.88	-0.52	-0.08	1.05
	23.38	23.03	-0.36	0.08	0.95
**Average**	23.39 ± 0.02	22.95 ± 0.11	-0.44 ± 0.11	0 ± 0.10	1 ± 0.07
**Sample #1 after CQ treatment**	25.05	23.89	-1.33	-0.89	1.85
	25.38	23.57	-1.65	-1.21	2.31
**Average**	25.21 ± 0.23	23.73 ± 0.23	-1.49 ± 0.23	-1.05 ± 0.23	2.06 ± 0.32
**Sample #2 after CQ treatment**	25.52	24.07	-1.94	-1.50	2.82
	26.49	24.80	-1.21	-0.76	1.70
**Average**	26.01 ± 0.69	24.44 ± 0.52	-1.57 ± 0.52	-1.13 ± 0.52	2.19 ± 0.79

Supplementary Table 12. Liquid chromatography multiple reaction monitoring (LC-MRM) parameters for the PvCRT selected peptide

Protein	Accession number	Peptide sequence	Q1 (m/z)	Q3 (m/z)	CE (V)	Precursor ion charge (z)	Product ion	Product ion charge (z)	Peptide type*
PvCRT	PVX_087980	IGNIILEK	450.3	786.5	20.0	2	y7	1	light
		IGNIILEK	453.4	792.6	20.0	2	y7	1	heavy
		IGNIILEK	450.3	389.4	20.0	2	y3	1	light
		IGNIILEK	453.4	395.4	20.0	2	y3	1	heavy
		IGNIILEK	450.3	276.1	20.0	2	y2	1	light
		IGNIILEK	453.4	282.1	20.0	2	y2	1	heavy

Peptide IGNIILEK was assayed by measuring the LC-MRM signal from three transitions. Parameters listed in the Table were used for optimum LC-MRM transitions. This peptide is encoded by the last 7 codons of exon 13 and the first codon of exon 14 from *pvcrt*.

*heavy: C13 labeled internal standard peptide; light: unlabeled peptide from parasite protein.

Supplementary Table 13. PvCRT protein levels as determined by liquid chromatography multiple reaction monitoring (LC-MRM) analyses

Sample	IS	PS	Transition 1	IS	PS	Transition 2	IS	PS	Transition 3	Average transition
NIH-1993 S×R unselected *Saimiri* 3022	9008	1073	0.119	4307	677	0.157	2695	685	0.254	0.138*
NIH-1993 S×R unselected *Aotus* 86355‡	6717	609	0.091	3591	448	0.1255	2100	751	0.362	0.108*
NIH-1993 S×R unselected *Aotus* 86435	11726	3611	0.308	5397	1653	0.306	4052	1134	0.280	0.298
NIH-1993 S×R unselected *Aotus* 86484	12282	2751	0.224	5504	1545	0.281	4407	1031	0.234	0.246
NIH-1993 S×R selected *Saimiri* 4469 B1	6676	666	0.100	3122	460	0.147	1994	963	0.483	0.124*
NIH-1993 S×R selected *Saimiri* 4469 B2	7442	105	0.014	3731	67	0.018	2308	99	0.043	0.016*
NIH-1993 S×R selected *Aotus* 86564 B1	5852	273	0.047	3029	169	0.056	1999	781	0.391	0.052*
NIH-1993 S×R selected *Aotus* 86564 B2	9517	957	0.101	4758	514	0.108	3353	473	0.141	0.117
NIH-1993 S×R selected *Saimiri* 4680	10540	962	0.091	4887	591	0.121	3297	413	0.125	0.112
NIH-1993 S×R selected *Aotus* 86349	8168	596	0.073	4044	398	0.098	3073	309	0.101	0.091
Chesson *Saimiri* 4429	15585	1935	0.124	7695	822	0.110	5181	896	0.173	0.117*
Chesson *Saimiri* 3877	12693	3327	0.262	5386	1516	0.281	4569	1278	0.280	0.275
Chesson *Aotus* 86354 B1	7154	160	0.022	3420	70	0.020	2614	589	0.225	0.021*
Chesson *Aotus* 86354 B2	8009	661	0.082	4483	163	0.036	2896	183	0.063	0.059*
AMRU-I *Aotus* 85840	11014	1765	0.160	4956	791	0.160	3549	651	0.183	0.168
AMRU-I *Saimiri* 3848	14024	1187	0.085	6319	421	0.067	4523	382	0.084	0.079
Indonesia-XIX *Aotus* 86489‡	9939	1842	0.186	4751	907	0.191	3248	574	0.177	0.185
Indonesia-XIX *Aotus* 86582	8297	1306	0.157	4337	635	0.146	2770	430	0.155	0.153

Each transition represents the integrated peak area ratio calculated from the internal standard (IS) and protein sample (PS) signals.

Average transition values are from two (*) or three acceptable transitions confirmed by a ‘ratio of transition ratios’ ≤ 1.5.

B1 and B2, biological replicates. ‡ Results obtained from two technical replicates.

Supplementary Table 14. Estimations of PvCRT concentration in magnetically-purified *P. vivax*-infected red blood cells

Sample	#iEMP	Volume dissolved (μl)	Volume used (μl)	Digestion volume (μl)	Injection volume (μl)	Dilution factor	Average transition	Final ratio	Total PvCRT (fMol)	PvCRT/iE (zMol)
NIH-1993 S×R unselected *Saimiri* 3022	1.6×10^{8}	70	40	63	15	7.35	0.138	1.01	101.43	0.61
NIH-1993 S×R unselected *Aotus* 86355[#]	9.1×10^{7}	140	30	86.2	15	26.82	0.108	2.90	289.66	3.17
NIH-1993 S×R unselected *Aotus* 86435	4.4×10^{8}	200	30	46.6	15	20.71	0.298	6.17	617.16	1.40
NIH-1993 S×R unselected *Aotus* 86484	5.3×10^{8}	200	30	55.1	15	24.49	0.246	6.02	602.45	1.13
NIH-1993 S×R selected *Saimiri* 4469 B1	1.4×10^{7}	70	30	49	15	7.62	0.124	0.94	94.49	6.60
NIH-1993 S×R selected *Saimiri* 4469 B2	7.4×10^{6}	30	30	44.6	15	2.97	0.016	0.05	4.75	0.68
NIH-1993 S×R selected *Aotus* 86564 B1	2.1×10^{7}	100	40	80	15	13.33	0.052	0.69	69.32	3.33
NIH-1993 S×R selected *Aotus* 86564 B2	6.0×10^{7}	100	20	30	15	10.00	0.117	1.17	117.00	1.94
NIH-1993 S×R selected *Saimiri* 4680	3.1×10^{7}	150	40	47	15	11.75	0.112	1.32	131.60	4.25
NIH-1993 S×R selected *Aotus* 86349	NA	150	40	66	15	16.50	0.091	1.50	150.15	NA
Chesson *Saimiri* 4429	1.7×10^{8}	60	40	15	15	1.50	0.117	0.18	17.55	0.10
Chesson *Saimiri* 3877	3.9×10^{8}	120	20	46	15	18.40	0.275	5.06	506.00	1.27
Chesson *Aotus* 86354 B1	2.2×10^{7}	100	30	70	15	15.56	0.021	0.33	32.68	1.46
Chesson *Aotus* 86354 B2	1.1×10^{8}	100	20	46.8	15	15.60	0.059	0.92	92.04	0.83
AMRU-I *Aotus* 85840	3.1×10^{8}	120	30	63	15	16.80	0.168	2.82	282.24	0.91
AMRU-I *Saimiri* 3848	4.7×10^{7}	70	30	57	15	8.87	0.079	0.70	70.07	1.49
Indonesia-XIX *Aotus* 86489[‡]	6.2×10^{8}	70	30	67.8	15	10.55	0.185	1.95	195.18	0.31
Indonesia-XIX *Aotus* 86582	1.6×10^{8}	100	25	41	15	10.93	0.153	1.67	167.23	1.01

Blood samples were assessed for parasitemia and parasite stage distribution; the number of cells were counted using an automated cell counter; each sample was passed through a MACS column to magnetically purify trophozoites, schizonts, and gametocytes, blood stages which have well developed food vacuole, where PvCRT may be expressed, and CQ may act. See Methods for details.

[‡] Results obtained from two technical replicates.

Supplementary Table 15. Repeat sequences in *pvcrt* 5'-UTR and intron 9 that are found in regulatory elements of other species

**Repeat**	**Number in 5'-UTR**		**Number in intron 9**		**Number in other *pvcrt* regions**	
	**NIH-1993-S**	**NIH-1993-R**	**NIH-1993-S**	**NIH-1993-R**	**NIH-1993-S**	**NIH-1993-R**
**TGAAGC**	6	12	6	4	2 (exons 2, 7)	2 (exons 2, 7)
**TGAGGC**	0	0	0	1	0	0
**TGAAGA**	2	2	8	8	1 (exon 2)	1 (exon 2)
**TGAGGA**	1	1	2	0	0	0
**TGAAGT**	4	3	0	0	0	0
**GCGAAAT (PB)**	1	1	0	0	0	0
**TCAAAC (DSR)**	2	2	0	0	0	0
**GAAGAAGAA (ESE)**	0	0	0	0	2 (exon 1; 3'-UTR)	2 (exon 1; 3'-UTR)
**AGAAA (LPE)**	0	0	2	0	0	0
**AAAG (Dof)**	1	1	13	11	4 (exons 2, 7, 14; intron 7)	4 (exons 2, 7, 14; intron 7)

TGAAGH repeat sequences (TGAAGC, TGAAGA, and TGAAGT) can modulate gene transcription when present in 5'-UTR, introns, and exons of *Arabidopsis*[9, 10, 11, 12]. Versions of these repeats with disruption of 'GAAG' core are shown in with the mutation in red. PB, Pausing Button sequence for RNA polymerase II in *Drosophila*[1]; DSR, Determinant of Selective Removal motif that recruits RNA elimination machinery for cell cycle stage-specific gene silencing in yeast[2]; ESE, Exon Splicing Enhancer motif, which regulates HIV type 1 splicing[3]; LPE, Late Pollen Element, responsible for pollen time specific expression of tomato genes[4], and also

observed in promoter regions of *Arabidopsis* genes[5]; Dof, a binding site for members of the DNA-binding with one finger transcription factor family, which is key for light and pathogen induced gene transcription in plants[6].

Supplementary Table 16. Sequences of oligonucleotide primers used in this study

Name of oligonucleotide primer	Nucleotide sequence from 5' to 3'
**MS334F**	GAAATGTAGATTTAAGTGCA
**MS334R**	TGTCACTTTGTCAAATAACA
**In9*pvcrt*F**	TGTGCGACGATTGCGAAGGA
**In9*pvcrt*R**	AGCAGCTGCGATTTGCCTCA
**MS283F**	TAGCTCTCCTAGCTCTAT
**MS283R**	CGTTTGAGCGCTAAAAAG
**MS33F**	CGATTCGTGCTATTTGCT
**MS33R**	CCTGCTACATATTTGGC
**MS1F**	TCAACTGTTGGAAGGGCAAT
**MS1R**	CTGTCTTTTGCTGCGTTTTTGTTTCTG
**MS35F**	TAACTGAGTCACCTCATCT
**MS35R**	TTACACGAGGTTTCCCTTT
**MS3F**	GAAGATCCTGTGGAGGAGCA
**MS3R**	CTGTCTTCTCCTTCGCTCCTTTCCTTT
**MS85F**	ATTCATAACGTAGCATATGT
**MS85R**	CAGTTTGGCTATCTACTAA
**MS12F**	AATGCGCATCCTATGTCTCC
**MS12R**	CTGTCTTCTGCTGTTGTTGTTGCTGCT
**MS52F**	ACATGTTGCATAGCTGGAA
**MS52R**	ACTGCACAGTGGTGAGTT
**MS15F**	TGTTTGCAAAGGAATCCACA
**MS15R**	CTGTCTTCGGCCAGATGAAAAGGATAA
**MS2F**	GAGCTAGCCAAAGGTTCAACA
**MS2R**	CTGTCTTTGGGGAGAGACTCCCTTTTC
**MS4F**	CGATTTACTGTTGACGCTGAA
**MS4R**	CTGTCTTCAAAGGAACATGCTCGATGA
**MS50F**	CAAAGGAACATGCTCGAT
**MS50R**	GAATTCTGAAGGAATTAGG
**MS5F**	CGTCCTCTATCGCGTACACA

**MS5R**	CTGTCTTAAAGGGAGAGGAGCGAAAAC
**MS41F**	AACTCGCGCTCGATTTCTT
**MS41R**	AGTTGAAGTACGGAGATTT
**MS38F**	AACAACGCAATGTGGATAA
**MS38R**	TTACGCTTAACTCATTCGT
**MS71F**	AAGGCCACATGTTCACCTTC
**MS71R**	ATGCTCATAAGAAAGGGGCA
**MS200F**	TTATTGCACAGCATTTTATG
**MS200R**	ATCAGCATTTCGATCAACA
**MS9F**	AGATGCCTACACGTTGACGA
**MS9R**	CTGTCTTGAAGCTGCCCATGTGGTAAT
**MS197F**	AGGTTATATTTTCCTTTCGA
**MS197R**	TAAGCGAGATATGCATACT
**MS196F**	ATCGTATGTGCGAAGTTAT
**MS196R**	AATTATGCTATACGACTTAC
**MS16F**	TGTTGTGGTTGTTGATGGTGA
**MS16R**	CTGTCTTGTCGGGGAGAACAACAACAT
**MsnMDRF**	CACTGGTTGAAGTGGTCCCA
**MsnMDRR**	GGCGTGGATTAGATTATGCA
**MS128F**	TTGAGGAAGTAATACAGGT
**MS128R**	AAGCTCAGCGACTACTTT
**MS116F**	AAATGCAAGATCCAAGAAAT
**MS116R**	GTCGCTCTTCATGTGGCA
**MS20F**	GCACAACAAATGCAAGATCC
**MS20R**	CTGTCTTGTGGCAGTGGCTCATCTTCT
**MS6F**	GGTTCTTCGGTGATCTCTGC
**MS6R**	CTGTCTTGGAGGACATCAACGGGATT
**MS17F**	CTAAATTAAGGGTATGAGAT
**MS17R**	TGCATACATAAATGCTTCAT
**MS7F**	TTGCAGAAAATGCAGAGAGC
**MS7R**	CTGTCTTAGGGTCTTCAGCGTGTTGTT
**MS209F**	GAGGTAAAAATGTAGCATG

**MS209R**	TTGTCAATTTTAGTAGACAC
**MS8F**	AGAGGAGGCAGAAATGCAGA
**MS8R**	CTGTCTTAGCCCCTTTGCGTTCTTTAT
**MS206F**	TCTTTATGTTGTACTGCTC
**MS206R**	ACCACTTACAAAAGTGTGA
**MS10F**	TTATCCCTGCTGGATGTGAA
**MS10R**	CTGTCTTTCCTTCAGGTGGGACTTGTT
**MS176F**	ATAATGGCGTCATCCTTCA
**MS176R**	TTCAGCATGCGCTGTTTAT
**MS183F**	AAGACGATTTACCAAAAACT
**MS183R**	GGATTTTTCGCTTATCATTT
**MS182F**	AAATGAATTCATGTAGTAGG
**MS182R**	TAAGCGGTTGTTACGTGAA
**pvcrt-RT-Fwd**	ATGTCCAAGATGTGCGACGAT
**pvcrt-RT-Rev**	CTGGTCCCTGTATGCAACTGAC
**pvSerylT-RNA-Fwd**	ACAACGCATTAGCAGCGAG
**pvSerylT-RNA-Rev**	ATCCCATGTTTGTTCCGCC

Supplementary References

1. Kwak H, Fuda NJ, Core LJ, Lis JT. Precise maps of RNA polymerase reveal how promoters direct initiation and pausing. *Science* **339**, 950-953 (2013).

2. Yamashita A, *et al.* Hexanucleotide motifs mediate recruitment of the RNA elimination machinery to silent meiotic genes. *Open Biol* **2**, 120014 (2012).

3. Staffa A, Cochrane A. Identification of positive and negative splicing regulatory elements within the terminal *tat-rev* exon of human immunodeficiency virus type 1. *Mol Cell Biol* **15**, 4597-4605 (1995).

4. Twell D, Yamaguchi J, Wing RA, Ushiba J, McCormick S. Promoter analysis of genes that are coordinately expressed during pollen development reveals pollen-specific enhancer sequences and shared regulatory elements. *Genes Dev* **5**, 496-507 (1991).

5. Casas-Mollano JA, Lao NT, Kavanagh TA. Intron-regulated expression of *SUVH3*, an *Arabidopsis Su(var)3-9* homologue. *J Exp Bot* **57**, 3301-3311 (2006).

6. Yanagisawa S. Dof domain proteins: plant-specific transcription factors associated with diverse phenomena unique to plants. *Plant Cell Physiol* **45**, 386-391 (2004).

7. Carlton JM, *et al.* Comparative genomics of the neglected human malaria parasite *Plasmodium vivax*. *Nature* **455**, 757-763 (2008).

8. Chattopadhyay R, *et al.* Establishment of an *in vitro* assay for assessing the effects of drugs on the liver stages of *Plasmodium vivax* malaria. *PLoS One* **5**, e14275 (2010).

9. Pertea M, Mount SM, Salzberg SL. A computational survey of candidate exonic splicing enhancer motifs in the model plant *Arabidopsis thaliana*. *BMC Bioinformatics* **8**, 159 (2007).

10. Ho LH, *et al.* Identification of regulatory pathways controlling gene expression of stress-responsive mitochondrial proteins in *Arabidopsis*. *Plant Physiol* **147**, 1858-1873 (2008).

11. Rose AB. Intron-mediated regulation of gene expression. *Curr Top Microbiol Immunol* **326**, 277-290 (2008).

12. Rose AB, Elfersi T, Parra G, Korf I. Promoter-proximal introns in *Arabidopsis thaliana* are enriched in dispersed signals that elevate gene expression. *The Plant cell* **20**, 543-551 (2008).
