# Supplementary material for: Plasmodium vivax chloroquine resistance links to pvcrt transcription in a genetic cross
Source: Nat Commun. 2019 Sep 20;10:4300. doi: 10.1038/s41467-019-12256-9 (PMC6754410; doi:10.1038/s41467-019-12256-9)
Supplement: Supplementary file 3 — Reporting Summary [file 41467_2019_12256_MOESM3_ESM.pdf]

nature research

Corresponding author(s): Thomas E. Wellems
Last updated by author(s): Aug 5, 2019

# Reporting Summary

Nature Research wishes to improve the reproducibility of the work that we publish. This form provides structure for consistency and transparency in reporting. For further information on Nature Research policies, see Authors & Referees and the Editorial Policy Checklist.

## Statistics

For all statistical analyses, confirm that the following items are present in the figure legend, table legend, main text, or Methods section.

| n/a | Confirmed | |
|---|---|---|
| ☐ | ☒ | The exact sample size (*n*) for each experimental group/condition, given as a discrete number and unit of measurement |
| ☐ | ☒ | A statement on whether measurements were taken from distinct samples or whether the same sample was measured repeatedly |
| ☐ | ☒ | The statistical test(s) used AND whether they are one- or two-sided<br>*Only common tests should be described solely by name; describe more complex techniques in the Methods section.* |
| ☐ | ☒ | A description of all covariates tested |
| ☐ | ☒ | A description of any assumptions or corrections, such as tests of normality and adjustment for multiple comparisons |
| ☐ | ☒ | A full description of the statistical parameters including central tendency (e.g. means) or other basic estimates (e.g. regression coefficient) AND variation (e.g. standard deviation) or associated estimates of uncertainty (e.g. confidence intervals) |
| ☐ | ☒ | For null hypothesis testing, the test statistic (e.g. *F*, *t*, *r*) with confidence intervals, effect sizes, degrees of freedom and *P* value noted<br>*Give P values as exact values whenever suitable.* |
| ☒ | ☐ | For Bayesian analysis, information on the choice of priors and Markov chain Monte Carlo settings |
| ☒ | ☐ | For hierarchical and complex designs, identification of the appropriate level for tests and full reporting of outcomes |
| ☒ | ☐ | Estimates of effect sizes (e.g. Cohen's *d*, Pearson's *r*), indicating how they were calculated |

*Our web collection on statistics for biologists contains articles on many of the points above.*

## Software and code

Policy information about availability of computer code

| | |
|---|---|
| Data collection | Prism/GraphPad and Excel were used to record parasitemia, genotype, qRT-PCR, and MRM data. |
| Data analysis | Data analysis for Figures 2a, 3a, and 3b used R Version 3.5.1. |

For manuscripts utilizing custom algorithms or software that are central to the research but not yet described in published literature, software must be made available to editors/reviewers. We strongly encourage code deposition in a community repository (e.g. GitHub). See the Nature Research guidelines for submitting code & software for further information.

## Data

Policy information about availability of data

All manuscripts must include a data availability statement. This statement should provide the following information, where applicable:
- Accession codes, unique identifiers, or web links for publicly available datasets
- A list of figures that have associated raw data
- A description of any restrictions on data availability

Targeted Illumina sequencing data of NIH-1993 S×R progeny LGS sample pairs are deposited in the NCBI Short Read Archive: SRR2148595-2148598, SRR2148600-2148607.

# Field-specific reporting

Please select the one below that is the best fit for your research. If you are not sure, read the appropriate sections before making your selection.

☒ Life sciences ☐ Behavioural & social sciences ☐ Ecological, evolutionary & environmental sciences

# Life sciences study design

All studies must disclose on these points even when the disclosure is negative.

| | |
|---|---|
| Sample size | Plasmodium vivax parasites cannot be cultivated by tissue culture methods, severely limiting studies of drug response and genetics. This and the requirement for nonhuman primate (NHP) infections are the major reasons why no P. vivax genetic crosses have been reported before. Since in vitro methods that were available for five previous Plasmodium falciparum genetic crosses could not be applied to P. vivax, we used Linkage Group Selection (LGS) analysis, developed from rodent systems, to compare allele prevalence in mixed populations of recombinant progeny before and after drug pressure (manuscript pp 6-7, lines 134-140). Accordingly, pools of blood stage recombinant P. vivax progeny were developed in 20 animals, since we know that phenotype and genotype data from a minimum of 15 segregants can support the mapping of a single locus determining drug response [Wellems et al. 1991 Proc Natl Acad Sci U S A. 88:3382-6]. |
| Data exclusions | All data available from the pairs of unselected vs. selected progeny (10 animals) were included in our analysis (no exclusions). Infections of NHPs that did not recrudesce could provide no selected progeny for paired analysis, as described in the text. |
| Replication | Individual samples of blood from NIH-1993 S×R-infected animals contained different populations of mixed recombinant progeny, each with their unique combinations of phenotypes and genotypes. The RT-PCR results from two separate samples of selected progeny from each of three monkeys show that the finding of upregulated transcription was replicated, a finding confirmed by the statistical analysis and calculated p value of 0.006. To meet replication/quality criteria in the proteomic studies, we required average values from two or three acceptable transitions that were confirmed by a 'ratio of transition ratios' ≤ 1.5' (Supplementary Table 13). |
| Randomization | See the comment above on unique populations of mixed recombinant progeny in the monkey blood. The main comparisons of groups of parasites was between the pre-treatment and post-treatment groups. The creation of the experimental groups was due to selection pressure of the chloroquine treatment in the host animals. Host animals in our study were picked from the available supply. Since this was no investigator allocation of units to groups, there was no randomization procedure required. |
| Blinding | All genomic DNA samples genotyped by microsatellite markers were performed and analyzed blindly. Host animals were not allocated into experimental groups, so no blinding of group membership was required. |

# Reporting for specific materials, systems and methods

We require information from authors about some types of materials, experimental systems and methods used in many studies. Here, indicate whether each material, system or method listed is relevant to your study. If you are not sure if a list item applies to your research, read the appropriate section before selecting a response.

## Materials & experimental systems

| n/a | Involved in the study | |
|---|---|---|
| ☐ | ☒ | Antibodies |
| ☐ | ☒ | Eukaryotic cell lines |
| ☒ | ☐ | Palaeontology |
| ☐ | ☒ | Animals and other organisms |
| ☒ | ☐ | Human research participants |
| ☒ | ☐ | Clinical data |

## Methods

| n/a | Involved in the study | |
|---|---|---|
| ☒ | ☐ | ChIP-seq |
| ☒ | ☐ | Flow cytometry |
| ☒ | ☐ | MRI-based neuroimaging |

## Antibodies

| | |
|---|---|
| Antibodies used | Monoclonal antibodies (MAb) against two variations of the Plasmodium vivax circumsporozoite protein (PvCSP): MAb VK210 (which recognizes the PvCSP common repeat DRAD/AGQPAG, BEI Resources, NIAID, NIH, catalog number MRA-184) and MAb VK247 (which recognizes a PvCSP variant repeat ANGAGNQPG, BEI Resources, NIAID, NIH, catalog number MRA-185) both MAbs were obtained from the Malaria Research and Reference Reagent Resource Center, BEI Resources, Manassas, VA, contributed by Elisabeth Nardin). Alexa Fluor 488 Goat anti-mouse IgG and Alexa Fluor 594 Goat anti-mouse IgG were obtained from Life Technologies). |
| Validation | These antibodies have been used in several malaria reports, but it was previously tested by co-authors of this manuscript in the following article:<br>Chattopadhyay, R. et al. Establishment of an in vitro assay for assessing the effects of drugs on the liver stages of Plasmodium vivax malaria. PLoS One 5, e14275 (2010). |

## Eukaryotic cell lines

Policy information about cell lines

| | |
|---|---|
| Cell line source(s) | Plasmodium vivax parasites in nonhuman primate erythrocytes:<br>P. vivax NIH-1993 was obtained from NIH archive cryopreserved samples from Aotus T308, May 1993. The following strains |

| | |
|---|---|
| | were obtained through BEI resources, NIAID, NIH: P. vivax AMRU-I (catalog number MRA-372), Indonesia-XIX (catalog number MRA-378), and Chesson (catalog number MRA-383). Parasite line NIH-1993 line was initially identified in NIH stocks as the CQ-sensitive Salvador-I strain. However, comparisons to the nuclear chromosome sequences and the mitochondrion genome of Salvador-I (http://www.vivaxmalaria.com/template_genomics.htm) showed polymorphisms that distinguish NIH-1993 and Salvador-I, including three dihydrofolate reductase mutations at codon positions 57, 99 and 173 (Presented in Supplementary Table 1). |
| Authentication | Cells lines were genotyped using DNA microsatellite markers. |
| Mycoplasma contamination | Cell lines were not tested for mycoplasma contamination. |
| Commonly misidentified lines (See ICLAC register) | *Name any commonly misidentified cell lines used in the study and provide a rationale for their use.* |

## Animals and other organisms

Policy information about studies involving animals; ARRIVE guidelines recommended for reporting animal research

| | |
|---|---|
| Laboratory animals | Aotus nancymaae (male and female, age range 3 – 7 years old), Saimiri boliviensis (male and female, age range 3 – 7 years old), and Pan troglodytes (female, 4 years old) were obtained from the National Institutes of Health (NIH) approved sources and housed in compliance with the Animal Welfare Act and the NIH Guide for the Care and Use of Laboratory Animals. |
| Wild animals | The study does not involve wild animals. |
| Field-collected samples | The study does not involve field-collected samples. |
| Ethics oversight | All animal procedures were performed in accordance with the National Institutes of Health Guidelines under protocols approved by Animal Care and Use Committees (ACUC) of the National Institute of Allergy and Infectious Diseases (NIAID) and Bioqual Inc. (Rockville, MD, USA). |

Note that full information on the approval of the study protocol must also be provided in the manuscript.
