# Supplementary material for: Plasmodium vivax chloroquine resistance links to pvcrt transcription in a genetic cross
Source: Nat Commun. 2019 Sep 20;10:4300. doi: 10.1038/s41467-019-12256-9 (PMC6754410; doi:10.1038/s41467-019-12256-9)
Supplement: Supplementary file 4 — Description of Additional Supplementary Files [file 41467_2019_12256_MOESM4_ESM.pdf]

Description of Additional Supplementary Files

File Name: Supplementary Software 1
Description: Statistical code and its description in three zipped files. The Rnw file produces a report of the analyses for Figures 2a,3a, and 3b using the Sweave system of the R computer language. Two PDF files provide further information about the software and how to use it to create the report.
