# Supplementary material for: Plasmodium vivax chloroquine resistance links to pvcrt transcription in a genetic cross
Source: Nat Commun. 2019 Sep 20;10:4300. doi: 10.1038/s41467-019-12256-9 (PMC6754410; doi:10.1038/s41467-019-12256-9)
Supplement: Supplementary file 5 — Supplementary Software 1 [file 41467_2019_12256_MOESM5_ESM.zip › 199166_2_supp_0_pwd7hc/Supplementary Software/Analyses_for_Figures_2a_3a_3b.pdf]

Supplementary Analysis and Code for Figures 2a, 3a, and 3b of "*Plasmodium vivax* chloroquine resistance links to pvcrt transcription in a genetic cross" by Sá, *et al.*

Summary

This supplement gives the analyses and code for Figures 2a (Section 2), 3a (Section 3), and 3b (Section 4). We begin with a brief description of the software used (Section 1).

1 How This File was Created and Where the R Code Resides

The `Analyses_for_Figures_2a_3a_3b.pdf` file for this supplement was created using the R computer language and the Sweave system. Therefore, this text and the R code that creates all the output for the pdf file, are both embedded within the `Analyses_for_Figures_2a_3a_3b.Rnw` file. The resulting pdf file may be recreated after installing the R software [`https://cran.r-project.org`], installing Rstudio [`http://www.rstudio.com`], installing the R package plyr, then using the "Compile PDF" option on the `Analyses_for_Figures_2a_3a_3b.Rnw` file.

2 Analyses for Figure 2a

In Figure 2a, if there was no change in the prevalence of the NIH-1993-R and NIH-1993-S between the before and after CQ treatment, then we would expect that the E value for each monkey and marker would be equally likely to be positive as to be negative. Under the null hypothesis model, we simulate the probability that we see k or greater markers where all 10 monkeys have the E value of the same sign, for $k = 1, 2$. It is hard to do the simulation because we do not know how correlated the E value for each marker will be to the other ones on the same chromosome. So instead of counting the amount of times each marker is either all positive E value or all negative E value, we count the number of times each chromosome has all its markers either all positive or all negative. For the simulation, we make two simplifying assumptions (1) all markers on the same chromosome will all have E values of the same sign, and (2) under the null hypothesis of no selection, the E values from different chromosomes are independent. If Assumption (1) is false, that will just decrease the power of our test to detect and effect, it will not cause the test to create more false postive signals (i.e., it will not inflate the type I error rate). So we really only need Assumption (2) to be able to use the p-values.

We only have 14 chromosomes. We can simulate the probability that 1 out of the 14 chromosomes will have either all 10 positive or all 10 negative E values.

We run a simulation with $1e+05$ replications. The simulated two-sided p-value for 1 or more chromosomes is $p_1^{(chrom)} = 0.02624$.

3 Analyses for Figure 3a

3.1 Decription of Data

We get the data from Supplementary Table 11, using the last column values: $2^{\Delta\Delta Ct}$, *pvcrt* transcription relative to its average transcription prior to CQ treatment. We use the average of the two values as the response (3rd row within each sample). So by definition, all values prir to CQ treatment are 1, and the values after CQ treatment represent fold change from values prior to CQ treatment.

We have 5 monkeys, with 2 monkeys measured once after CQ treatment and 3 monkeys measured twice after CQ treatment.

```
         animal  species prepost sampleNum resp
1   Aotus.85986    aotus     pre         0 1.00
2   Aotus.85986    aotus    post         1 1.13
3   Aotus.85986    aotus    post         2 6.34
4   Aotus.86121    aotus     pre         0 1.00
5   Aotus.86121    aotus    post         1 3.74
6   Aotus.86121    aotus    post         2 8.02
7   Aotus.WR454    aotus     pre         0 1.00
8   Aotus.WR454    aotus    post         1 1.72
9  Saimiri.4919 saimiri     pre         0 1.00
10 Saimiri.4919 saimiri    post         1 2.87
11 Saimiri.5081 saimiri     pre         0 1.00
12 Saimiri.5081 saimiri    post         1 2.06
13 Saimiri.5081 saimiri    post         2 2.19
```

3.2 Analysis

If each monkey was measured only once after CQ treatment, then we could take the log of the fold change for all 5 monkeys and perform a t-test. Taking the mean of the log(fold-change) and expontiating gives the geometric mean of the fold-change, and exponentiating the confidence limits gives a confidence interal on the fold-change.

Since we have repeated after-treatment measurements on 3 monkeys, we use within-cluster resampling to perform the analysis (see e.g., Follmann, PRoschan, and Leifer, 2003). Similar to the t-test method described above, we work on the log-transformed responses and back-transform the results. Instead of using a normal distribution for inferences, we use the t distribution with 4 degrees of freedom, so that if there was no repeated measurements, the results would be equivalent to the t-test methods described in the previous paragraph.

Here are the fold-change estimate, the associated 95% confidence interval, and the two-sided p-value for testing for a difference from a fold-change of 1.

```
    FoldChange         lowerCL.FC         upperCL.FC two.sided.p.value
         2.402              1.531              3.771             0.006
```

4 Analyses for Figure 3b

4.1 Summary

This section includes analyses using the log-transformed data and the non transformed data. The better analysis uses the log-transformed data, so that all the confidence interals have positive limits.

If we do not log-transform the data, then some of the confidence intervals have negative lower limits, pointing out the lack of appropriateness of the t-test on the untransformed data.

4.2 The Data

Here are the data:

```
      y                  group
1  0.61 NIH-1993 SxR unselected
2  3.17 NIH-1993 SxR unselected
3  1.40 NIH-1993 SxR unselected
4  1.13 NIH-1993 SxR unselected
5  6.60   NIH-1993 SxR selected
6  0.68   NIH-1993 SxR selected
7  3.33   NIH-1993 SxR selected
8  1.94   NIH-1993 SxR selected
9  4.25   NIH-1993 SxR selected
10 0.10                Chesson
11 1.27                Chesson
12 1.46                Chesson
13 0.83                Chesson
14 0.91                 AMRU-I
15 1.49                 AMRU-I
16 0.31          Indonesia-XIX
17 1.01          Indonesia-XIX
```

4.3 Analysis on Log-scale (Fold-change)

We perform a one-sample t-test on the log-transformed responses for each group, and then back-transform the statistics (so that the estimate is the geometric mean). The plots are done on a log-scale.

Here are the statistics for each group:

	group	estimate	lowerCL	upperCL
1	AMRU-I	1.1644312	0.0507726125	26.705342
2	Chesson	0.6263379	0.0861778312	4.552205
3	Indonesia-XIX	0.5595534	0.0003082877	1015.609819
4	NIH-1993 SxR selected	2.6190124	0.8848983151	7.751428
5	NIH-1993 SxR unselected	1.3225098	0.4475391409	3.908110

We next perform the two-sample t-test (Welch's version). The effect statistic is fold-change, which is the ratio of geometric mean of the NIH-1993 SxR selected over the geometric mean of the NIH-1993 SxR unselected.

Fold.Change	lowerCL	upperCL	twosided.pvalue
1.9803350	0.5813655	6.7457163	0.2289232

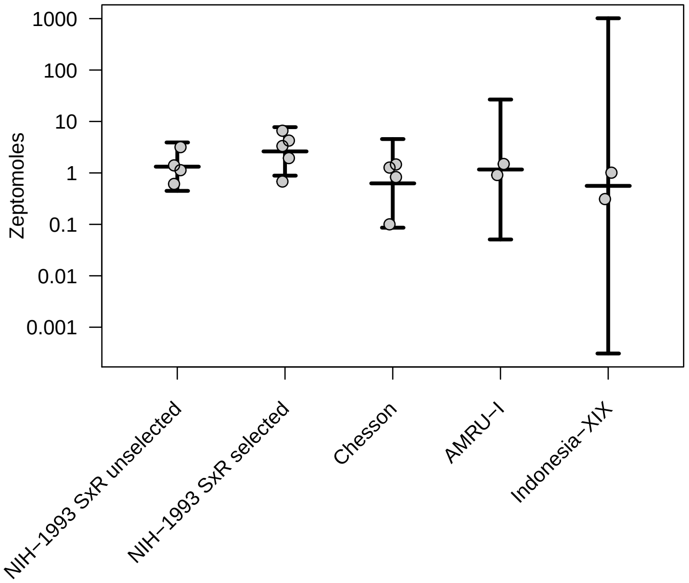

Figure 1: Middle lines are geometric means, the lower and upper bars are the 95% confidence intervals using a one-sample t-test on the log-transformed responses.

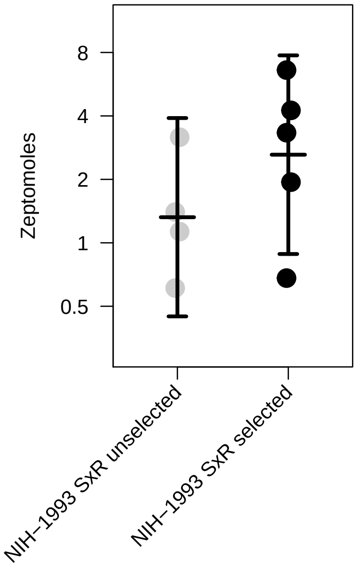

Figure 2: Middle lines are geometric means, the lower and upper bars are the 95% confidence intervals using a one-sample t-test on the log-transformed responses.

4.4 Analysis on Arithmetic scale

Here are the statistics on the untransformed data (so the estimate is the usual mean).

	group	estimate	lowerCL	upperCL
1	AMRU-I	1.2000	-2.48479937	4.884799
2	Chesson	0.9150	-0.04612162	1.876122
3	Indonesia-XIX	0.6600	-3.78717166	5.107172
4	NIH-1993 SxR selected	3.3600	0.54979039	6.170210
5	NIH-1993 SxR unselected	1.5775	-0.19056661	3.345567

Here are the results of the two-sample t-test (Welch's version). Here the effect estimate is the difference in means.

Diff.in.Means	lowerCL	upperCL	twosided.pvalue
1.7825000	-1.0379673	4.6029673	0.1732408

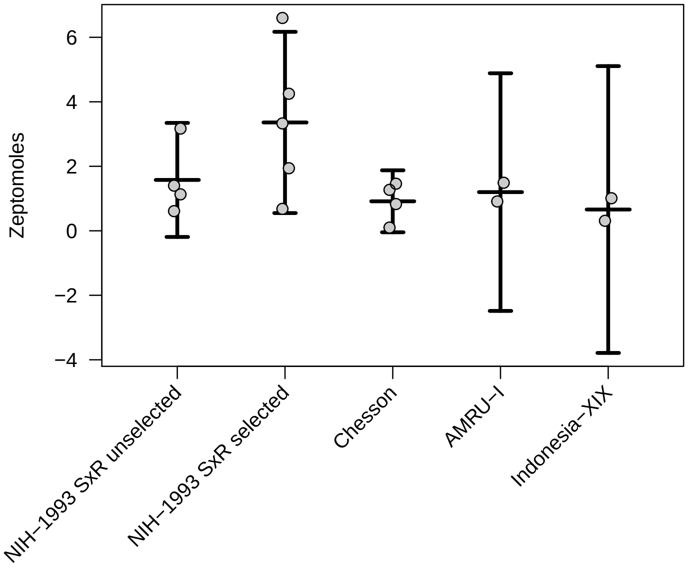

Figure 3: Untransformed data: Not Recommended (notice negative lower confidence limits)

References

Follmann, D, Proschan, M, and Leifer, E. (2003). Multiple outputation: inference for complex clustered data by averaging analyses from independent data. 59: 420-429.
