# Supplementary material for: Plasmodium vivax chloroquine resistance links to pvcrt transcription in a genetic cross
Source: Nat Commun. 2019 Sep 20;10:4300. doi: 10.1038/s41467-019-12256-9 (PMC6754410; doi:10.1038/s41467-019-12256-9)
Supplement: Supplementary file 5 — Supplementary Software 1 [file 41467_2019_12256_MOESM5_ESM.zip › 199166_2_supp_0_pwd7hc/Supplementary Software/Description of files.pdf]

The analyses were done using the R computer language, with the Sweave system used to create the reproducible document as a pdf file through the Rstudio system. The Analyses_for_Figure_2a_3a_3b.pdf file was created using the Analyses_for_Figure_2a_3a_3b.Rnw file and that system. See Section 1 of the pdf file for more details.
